# Circular and Long Non-Coding RNAs in Cancer Metabolism: Dual Perspective of Biomarkers and Therapeutic Targets

**DOI:** 10.3390/ncrna12020011

**Published:** 2026-03-19

**Authors:** Francesca Pia Carbone, Stefania Hanau, Nicoletta Bianchi

**Affiliations:** 1 Department of Translational Medicine, University of Ferrara, 44121 Ferrara, Italy; francescapia.carbone@unife.it; 2 Department of Neuroscience and Rehabilitation, University of Ferrara, 44121 Ferrara, Italy; stefania.hanau@unife.it

**Keywords:** non-coding RNAs, circular RNA, metabolites, cancer

## Abstract

**Background/Objectives**: Metabolic reprogramming is a hallmark of cancer, enabling tumor cells to sustain proliferation, survive under metabolic stress, and develop therapeutic resistance. While oncogenic signaling pathways regulating cancer metabolism have been extensively studied, increasing evidence indicates that non-coding RNAs (ncRNAs) play essential roles in coordinating metabolic adaptation. This review aims to synthesize current knowledge on long non-coding RNAs (lncRNAs) and circular RNAs (circRNAs) as important but relatively less characterized regulators of cancer metabolic adaptation and discuss their potential as biomarkers and therapeutic targets. **Methods**: We analyzed their roles across multiple types of cancer, prioritizing studies that integrate ncRNA profiling with metabolomics and mechanistic investigations, with particular attention to their diagnostic, prognostic, and predictive value. **Results**: LncRNAs and circRNAs regulate major metabolic pathways, including glycolysis, mitochondrial function, glutaminolysis, lipid metabolism, and redox balance. They act through transcriptional and epigenetic mechanisms, protein scaffolding, peptide encoding, and miRNA sponging, frequently converging on key regulators such as HIF-1α, c-Myc, p53, AMPK, and mTOR. However, many reported associations remain largely correlative, with limited integration of quantitative metabolic flux analyses and insufficient validation in physiologically relevant models. **Conclusions**: Although lncRNAs and circRNAs constitute an important context-dependent regulatory layer linking oncogenic signaling to metabolic reprogramming, future studies should combine ncRNA perturbation with stable isotope tracing, fluxomics, spatial metabolomics, long-read sequencing, and single-cell approaches to define causal and spatially resolved metabolic functions. Such integrative strategies may improve biomarker development and support ncRNA-informed, metabolism-oriented therapeutic interventions.

## 1. Introduction

In biology and medicine, the non-protein-coding genome is gaining increasing attention for its translational potential, particularly in the field of biomarker discovery [1]. Large-scale transcriptomic analyses indicate that a substantial portion of the human genome is transcribed, whereas only approximately 1–2% encodes protein-coding genes; the remaining transcripts are generally referred to as non-coding RNAs (ncRNAs) [2]. Non-coding RNAs (ncRNAs) are classified according to their length and structure. They include small ncRNAs (<200 nucleotides), such as miRNAs, piRNAs, and snRNAs/snoRNAs, and long non-coding RNAs (lncRNAs), defined as transcripts longer than 200 nucleotides without protein-coding potential. Circular RNAs (circRNAs) constitute a distinct class of covalently closed RNA molecules generated by back-splicing and lacking 5′ caps and 3′ poly(A) tails. In this review, we focus specifically on lncRNAs and circRNAs [1].

NcRNAs regulate gene expression and cellular pathways through diverse molecular mechanisms [3], acting as key modulators of transcriptional and post-transcriptional processes [4]. Their high tissue specificity, dynamic expression patterns, and remarkable stability, particularly in biofluids, make ncRNAs attractive candidates as cancer biomarkers. These molecules are involved in various biological processes, including development, differentiation, and disease, with a prominent role in cancer initiation and progression [1]. A growing body of evidence links ncRNA dysregulation to tumorigenesis, metastatic spread, and therapeutic resistance, highlighting their potential as diagnostic, prognostic, and predictive biomarkers.

The clinical utility of ncRNAs is further supported by their suitability in both liquid biopsy and tissue-based diagnostic platforms [5]. NcRNAs can function either as oncogenes or tumor suppressors, thereby reflecting tumor biology and disease aggressiveness. For example, specific lncRNAs promote cancer progression by regulating key oncogenic signaling pathways, while others exert tumor-suppressive effects by inhibiting tumor growth and metastasis [6]. Overall, these characteristics make ncRNAs promising candidates for improving cancer detection, patient stratification, and clinical decision-making.

This review focuses on the role of lncRNAs and circRNAs in metabolic pathways that are frequently dysregulated in cancer. MiRNAs, although representing a major class of ncRNAs, are excluded to maintain a defined scope, as their involvement in cancer metabolism has been extensively reviewed elsewhere. MiRNAs are discussed only when functionally relevant to the regulatory interplay with lncRNAs or circRNAs. With this aim, we surveyed the recent literature using the following search strategy and Query: ((“Non coding RNA” OR ncRNA[MeSH Terms]) AND metabolomic AND (Cancer[MeSH Terms])) NOT (miRNA OR MicroRNA) NOT (Review[pt]) NOT (Meta-analysis[pt]) NOT (“Systematic Review”[pt]). This search yielded 93 research articles. However, after a careful analysis of the manuscripts, not all were relevant to the scope of this review. Articles were included if they reported experimental evidence supporting the involvement of lncRNAs or circRNAs in cancer cell metabolism or metabolic adaptation, as well as studies identifying them as potential therapeutic targets. To ensure comprehensive coverage, the initial database search was supplemented by manually screening recent publications (within the last five years) and relevant references cited in selected articles.

### 1.1. Focusing on lncRNAs in Cancer

LncRNAs regulate gene expression at epigenetic, transcriptional, and post-transcriptional levels [1], and growing evidence supports their therapeutic potential in oncology [7]. At the molecular level, they interact with chromatin-modifying complexes and genomic DNA to shape chromatin states and the activity of regulatory elements [8,9,10,11,12]. Recent systematic analyses have further identified cancer-dysregulated lncRNAs with G-quadruplex (G4)-forming potential, highlighting structure-dependent regulatory mechanisms. Their altered expression and structural features may promote malignancy [13]. In the CanLncG4 dataset, several G4-bearing lncRNAs, including *GSEC*, *REG1CP*, *LU-CAT1*, *NEAT1*, and *HOTAIR*, are recurrently altered across multiple cancer types [13]. These transcripts may engage in G4-mediated interactions with RNA-binding proteins and epigenetic regulators, thereby influencing chromatin dynamics and transcriptional programs. Indeed, oncogenic lncRNAs such as *HOTAIR*, *MALAT1*, *LINC00152*, and *SNHG1* promote tumor initiation, progression, and drug resistance by recruiting histone-modifying complexes, including Polycomb Repressive Complex 2, and reshaping epigenetic landscapes [4,14,15]. Prototypical examples, including *XIST* and *NEAT1*, further illustrate the central role of lncRNAs in chromatin-based regulation of cancer-associated gene expression [16].

Reflecting their context-dependent expression patterns and regulatory versatility [17,18,19], lncRNAs extend their activity beyond chromatin-level control by modulating mRNA stability, splicing, localization, and translation. They achieve this through interactions with target transcripts by acting as miRNA decoys and sponges, thereby attenuating miRNA-mediated repression [20,21,22,23,24,25,26]. For example, *LINC00511* enhances gastric cancer cell proliferation and migration by targeting the tumor suppressor miR-765 [27], while *UCA1* promotes bladder cancer invasion and metastasis through miR-145 sponging and activation of the PI3K/AKT pathway [28]. Several lncRNAs also display marked cancer specificity [29]. *Lnc-SLC2A12-10:1*, detected in plasma-derived exosomes from patients with gastric cancer, reduces malignant features via miR-105-5p and miR-150-3p modulation. In hepatic cancer, lncRNA *PCNAP1* promotes tumor progression through miR-154/PCNA/HBV and miR-340-5p-dependent mechanisms and has also been implicated in breast cancer as a competitive endogenous RNA (ceRNA). Similarly, lncRNA *CDC6* functions as a ceRNA, regulating the expression of Cell Division Cycle 6 in breast cancer and participating in the regulatory *LINC01088*/microRNA-22/CDC6 network in prostate cancer, under the control of PI3K/AKT signaling cascade.

LncRNA *BCRT1* further exemplifies oncogenic activity across tumor types. It modulates cell cycle progression in cervical cancer via miR-432-5p and C-C Motif Chemokine Receptor 7, promotes metastasis in breast cancer under hypoxic conditions through Hypoxia-inducible factor 1α (HIF-1α)-associated mechanisms involving miR-1303 and Polypyrimidine Tract Binding Protein 3, and stimulates epithelial-to-mesenchymal transition (EMT) in osteosarcoma by regulating Fibroblast Growth Factor 7 expression. Likewise, lncRNA *IGFBP4-1* correlates with metastatic progression in lung cancer by modulating ATP production and the Warburg effect, and its oncogenic role in bladder urothelial carcinoma is abrogated by inhibition of the JAK/STAT pathway [29]. More broadly, lncRNAs operating within STAT signaling represent critical regulatory nodes, as their activity can directly undermine anti-tumor immune responses [30].

In addition, many lncRNAs contribute to cancer progression not only by modulating signaling pathways but also by directly reshaping cellular metabolism. The nuclear lncRNA *LETS1* amplifies TGFβ–SMAD signaling, promoting metastatic behavior in lung and breast cancer [31]. Because TGFβ-driven EMT requires coordinated shifts in glycolysis, glutamine utilization, and lipid metabolism, *LETS1* reinforces the metabolic adaptations that sustain migratory and invasive phenotypes, thereby linking lncRNA activity to metabolism-driven cellular plasticity.

*KB-1460A1.5* can be cited as a prime example of tumor-suppressive lncRNA. It is downregulated in gliomas and its overexpression triggers ferroptosis, an iron- and lipid peroxidation-dependent form of cell death, by inhibiting the mTOR/SREBP-1/SCD1 axis. This reduces the production of monounsaturated fatty acids, which normally protect cells from oxidative lipid damage, highlighting how lncRNAs can modulate lipid metabolism to sensitize tumor cells to oxidative stress.

LncRNAs further regulate redox metabolism, impacting therapy resistance. Glutathione (GSH) degradation-inhibiting lncRNA (*GDIL*), upregulated in platinum-resistant colorectal and ovarian cancers (OCs), enhances intracellular GSH levels by inhibiting its degradation via CHAC1 suppression [32]. This maintains redox balance under chemotherapy-induced oxidative stress, allowing cancer cells to survive platinum treatment. *GDIL* thus exemplifies how lncRNAs can modulate metabolic pathways that buffer Reactive Oxygen Species (ROS), directly contributing to chemoresistance.

In the study by Liu et al. [33], seven Mitochondrial Permeability Transition-Driven Necrosis-Related lncRNAs (*MPTDNR* lncRNAs) were identified in hepatocellular carcinoma (HCC) through a comparative analysis of tumor and adjacent non-tumor tissues. Mitochondrial permeability transition (MPT) involves the loss of inner mitochondrial membrane barrier function and, when dysregulated, may induce a form of programmed cell death implicated in tumor progression, therapeutic response, and prognosis. In this context, the authors identified a signature of seven lncRNAs (*PICSAR*, *AC025176.1*, *AC016405.3*, *LINC02313*, *AP002387.1*, *AC004687.1*, and *AL451069.3*) whose expression levels were significantly altered in HCC tissues and also correlated with patient prognosis. Among these, *LINC02313* emerged as a key prognostic and functional lncRNA, as its expression was markedly elevated in patients with the poorest outcomes and reduced in those with more favorable prognoses. High *LINC02313* expression was also associated with immune suppression, treatment resistance, and an immunologically “cold” tumor phenotype, characterized by reduced PD-L1 expression and a limited response to immune checkpoint inhibitors. Notably, the oncogenic role of *LINC02313* was further validated using a liver cancer organoid model, strengthening the translational relevance of these findings and supporting the potential of *LINC02313* as both a prognostic biomarker and a therapeutic target in HCC [33].

These examples indicate that lncRNAs act as key metabolic regulators in cancer, controlling fatty acid desaturation, nutrient utilization, and redox homeostasis, thereby shaping ferroptotic vulnerability, EMT-associated metabolic adaptation, and therapy resistance.

### 1.2. Focusing on circRNAs in Cancer

CircRNAs exert regulatory functions including miRNA sponging, protein interactions, and, in some cases, peptide encoding [1]. They are covalently closed single-stranded ncRNAs generated by back-splicing, in which the 3′ end of a pre-mRNA ligates to its 5′ end, producing exon- and/or intron-containing circular transcripts distinct from their linear counterparts (linRNAs) [34,35,36]. Two major categories have been described: intronic circRNAs, mainly nuclear (including some derived from tRNA), and exon–intron circRNAs [37,38,39,40]. Their circular structure confers resistance to exonuclease-mediated degradation and enhances the stability of these molecules [41,42].

Functionally, circRNAs regulate gene expression through transcriptional control, binding DNA or RNA, sequestration of miRNAs, and protein interactions [35,43,44,45]. Although they predominantly accumulate in the cytoplasm [46], nuclear circRNAs can interact with DNA, U1 small nuclear ribonucleoproteins, small nuclear RNAs, and RNA polymerase II, or form circR-loops, thereby modulating transcription and splicing [47]. The formation of RNA–DNA hybrids has been more extensively documented for lncRNAs than for circRNAs. Some circRNAs can recruit transcriptional activators to promote or enhance their nuclear import, whereas others interfere with transcription factor activity, affecting epigenetic and splicing mechanisms [48,49,50].

In the cytoplasm, circRNAs frequently act as miRNA sponges or competitive endogenous RNAs (ceRNAs), sequestering miRNAs and preventing their binding to target mRNAs, thereby increasing mRNA stability and expression, in some cases in an Argonaute-dependent manner [51,52]. Given the complexity of circRNA–miRNA networks in cancer, computational graph-based models have facilitated the identification of biologically relevant interactions by integrating higher-order network relationships and propagation strategies [53]. For instance, *circSLC26A4* and *circEPSTI1* contribute to cervical cancer progression through ceRNA-mediated mechanisms [54,55]. Beyond miRNA regulation, circRNAs interact with RNA-binding proteins (RBPs) and other regulatory factors, modulating protein stability, localization, ubiquitination, phosphorylation, and activity [56,57,58,59,60]. By acting as scaffolds, decoys, or stabilizing elements, circRNA–protein complexes influence transcriptional regulation, signal transduction, metabolic adaptation, stress responses, and oncogenic signaling [61,62,63,64]. CircRNAs may also facilitate the formation of ternary protein complexes [62], alter subcellular localization of binding partners [63,64], or associate with stress granules and participate in phase separation processes [40,65].

A subset of circRNAs is translated through cap-independent mechanisms, including internal ribosome entry sites, CU-rich elements, or m6A-modified start codons, and may undergo rolling circle translation [66]. CircRNA-derived peptides, frequently identified in tumors [39,67,68], modulate oncogenic pathways. For example, the E-cadherin-derived protein variant C-E-Cad, encoded by *circ-E-Cad* in glioblastoma, associates with the CR2 domain of EGFR and activates downstream signaling even in the absence of canonical ligands [69]. In gastric cancer, C-E-Cad activates the PI3K/AKT pathway and promotes oncogenic phenotypes, although supporting data are still limited [70].

CircRNAs display tissue- and development stage-specific expression patterns and are detectable in peripheral blood, supporting their diagnostic and prognostic relevance [40,71,72,73,74,75]. CircRNAs functioning as oncogenes or tumor suppressors are frequently deregulated in cancer, thereby reinforcing their relevance as diagnostic and prognostic biomarkers, as well as potential therapeutic targets [7,76,77]. For example, *circPVT1* is upregulated in various cancers and promotes tumor growth and metastasis by regulating the expression of target genes [78], whereas *circHIPK3* is downregulated in several tumor types and exerts tumor-suppressive effects by inhibiting cell proliferation and inducing apoptosis [79]. Additionally, *circ_0067934* drives pancreatic cancer progression via miR-1324 sponging and activation of the Wnt/β-catenin pathway [80], while *circ_0008450* promotes gastric cancer invasion and metastasis by sequestering miR-422a and upregulating the oncogene SOX4 [81]. Another example in gastric cancer is *circPGD*, which is markedly upregulated in tumor tissues. Mechanistically, *circPGD* promotes tumor progression by acting as a sponge for miR-16-5p, thereby derepressing ABL2 expression, and by encoding a novel peptide (PGD-219aa) derived from the phosphogluconate dehydrogenase *locus*. Through these dual functions, *circPGD* links oncogenic signaling with pentose phosphate pathway-associated metabolic regulation, supporting NADPH production, redox homeostasis, and anabolic metabolism during gastric carcinogenesis [35,82].

CircRNAs may serve as drug targets when they play a pathogenetic role or contribute to immunotherapy resistance [73,83], or as therapeutic agents when they exert disease-suppressive or immunostimulatory effects [84]. In addition to their intracellular roles, exosomal circRNAs are increasingly recognized as mediators of drug resistance. Packaged within tumor-derived extracellular vesicles (EVs), they can be transferred to recipient cancer cells or stromal components of the tumor microenvironment, where they modulate therapy response through miRNA sponging, regulation of signaling pathways, and control of apoptosis, autophagy, and EMT, thereby promoting metabolic adaptation, redox homeostasis, and cell survival [85]. Their high stability within EVs and detectability in body fluids underscore their dual relevance as drivers of therapeutic resistance and as non-invasive biomarkers for monitoring treatment response and disease progression. In colorectal cancer, lncRNAs and circRNAs operate within ceRNA networks that regulate drug transport, DNA damage response, apoptosis, and metabolic adaptation, thereby influencing chemosensitivity through PI3K/AKT, Wnt/β-catenin, p53, and mTOR signaling pathways [86]. These principles are exemplified in studies of oxaliplatin sensitivity: *circLRCH3* promotes resistance by sequestering miR-383-5p and activating FGF7; *circ0032821* enhances chemoresistance via the miR-515-5p/SOX9 axis; *circPDIA3* limits pyroptosis through interaction with the GSDME-C domain; *hsa_circ0071589* promotes cancer stemness through miR-133b/SOX13; conversely, *circPDE4D* acts as a tumor suppressor, and its downregulation facilitates resistant phenotypes [87]. These examples illustrate how circRNAs integrate oncogenic signaling with metabolic rewiring, ROS management, and stress-adaptive pathways, supporting their potential value as predictive biomarkers and therapeutic targets to overcome oxaliplatin resistance.

CircRNAs are also important regulators of tumor immunity and immunotherapy response: they modulate immune checkpoint expression, cytokine signaling, antigen presentation, and immune cell differentiation through miRNA sponging, interaction with RNA-binding proteins (RBPs), or peptide encoding, and their dysregulation has been linked to resistance or sensitivity to immune checkpoint inhibitors [88].

Recent advances have also highlighted the potential of circRNAs as a novel platform for cancer vaccine development [89]. Owing to their covalently closed structure, circRNAs exhibit enhanced stability, prolonged antigen expression, and reduced susceptibility to exonuclease degradation compared with linRNAs, making them attractive candidates for vaccine applications. CircRNA-based cancer vaccines can be engineered to encode tumor antigens or neoantigens and have been shown to efficiently activate antigen-presenting cells, promote cytotoxic T cell responses, and stimulate durable anti-tumor immunity. Interestingly, circRNA vaccines can be designed to integrate immunostimulatory elements, thereby enhancing innate immune activation while minimizing excessive inflammatory responses. These features position circRNA-based vaccines as a promising and flexible immunotherapeutic strategy, with potential applications in personalized cancer vaccination and combination therapies aimed at overcoming immune evasion.

With respect to the immune system, these molecules exert a dual function. In normal conditions, the cell-specific expression of circRNAs is reported in human hematopoietic progenitors and in M1 and M2 macrophage polarization. Chiefly, lymphocytes had the highest abundance of circRNAs derived from immunoglobulin genes [90,91,92]. CircRNAs have been shown to exert a wide range of functions in the immune system, including the regulation of innate immune signaling pathways such as the double-stranded RNA (dsRNA)-activated protein kinase in peripheral blood mononuclear cells, the modulation of pro-inflammatory cytokine expression (e.g., IL-6) in macrophages, and the control of immune cell fate by promoting macrophage senescence or apoptosis [93,94]. In this way, they participate in immune responses against tumors [95,96] and in immune inhibition mediated by cancerous cells themselves.

## 2. lncRNAs and circRNAs as Players in Cancer Metabolism and Metabolic Regulation

Metabolic reprogramming is a recognized hallmark of cancer. In this context, our work aims to provide an integrated overview of the role of lncRNAs and circRNAs in metabolic pathways. We will critically examine how these molecules contribute to the regulation of metabolic processes.

LncRNAs have recently emerged as important modulators of metabolic reprogramming in tumors, influencing mitochondrial function, glucose and lipid metabolism, and expression of engaged enzymes. CircRNAs, which have recently emerged as a distinct class of ncRNAs, are increasingly implicated in metabolic control, acting via miRNA sponging, protein interactions, and regulation of enzyme activity.

Overall, the diverse and increasingly recognized roles of these ncRNAs could represent a dual strategy to control the metabolic adaptation of cancer cells.

### 2.1. Integration of ncRNA Profiling and Metabolomics for Early Cancer Detection

The integration of ncRNA profiling with metabolomic analyses is increasingly recognized as a powerful strategy for early cancer detection and patient stratification. Different classes of ncRNAs contribute to cancer diagnosis through their tumor-specific expression patterns, functional involvement in metabolic reprogramming, and high stability in tissues and biofluids. Several ncRNAs discussed in this review, such as *MALAT1*, *PVT1*, *H19*, and *NEAT1*, have been identified as circulating or tissue-based biomarkers whose expression correlates with alterations in glucose, lipid, and amino acid metabolism. Large-scale ncRNA screening approaches, enabled by next-generation sequencing and transcriptomic profiling, have led to the identification of ncRNA signatures capable of distinguishing cancerous from non-cancerous tissues and predicting disease progression [97]. Recent advances include machine learning-assisted, renewable, and polarity-switchable photoelectrochemical biosensors that enable intelligent and highly sensitive circRNA detection, highlighting the potential of integrated AI-based platforms for circRNA-driven cancer diagnosis [98]. When combined with metabolomic readouts, ncRNA-based screening provides complementary molecular information that enhances diagnostic sensitivity and specificity, supporting the development of integrated, non-invasive strategies for early cancer detection. Firstly, accurate detection of ncRNA expression in pathogenic tissues requires optimized methods of isolation and sequencing [99]. Secondly, a variety of technologies enable their detection and quantification across different tissue types and preservation, including fresh-frozen and fixed samples, and applied technologies encompassing next-generation sequencing, hybridization-based approaches, and PCR-based assays. Furthermore, their detectability in extracellular and cellular compartments, such as extracellular vesicles and body fluids, including urine, saliva, cerebrospinal fluid, synovial fluid, placenta, and breast milk, highlights their suitability for liquid biopsy applications [7,100,101,102,103]. This paves the way for their integration with metabolomics studies.

For example, Hao et al. [104] integrated serum metabolomics with lncRNA profiling in endometrial cancer (EC) to identify non-invasive diagnostic biomarkers. Their analysis revealed pronounced deregulation of lipid and amino acid metabolism, likely reflecting membrane remodeling and alterations in pathways associated with tumor growth and progression. During the transition from endometrial atypical hyperplasia to stage I and stage III EC, metabolites exhibited dynamic changes; notably, linoleic acid levels increased continuously, whereas 4-chlorophenol (environmental exposure metabolite), glycyl-L-proline, and mannitol steadily decreased. These alterations suggest that these metabolites may serve as markers of disease progression, from precancerous lesions to malignant transformation. In parallel, lncRNAs mirrored these metabolic changes and are proposed as regulators of cancer metabolism, implicating them in EC pathogenesis. *LINC00511*, *PVT1*, and *IQCH-AS1* displayed the most significant differences between hyperplasia lesions and progressive disease, whereas in the dysplasia group, *MALAT1* and *CARMN* were downregulated, and *LINC00648*, *BISPR*, *LINC01534*, and *LINC00930* were upregulated [105]. Pathway analysis indicated enrichment of both differentially accumulated metabolites and expressed lncRNAs in lipid metabolism, underscoring their critical role in EC development. Notably, *PVT1* is a well-characterized oncogene regulated by the tumor suppressor p53, influencing c-Myc expression and promoting tumorigenesis [106]. Likewise, *MALAT1* is associated with EMT, emphasizing its relevance to EC progression [106].

The integration of metabolomics and lncRNA profiling offers a promising approach for the early detection of endometrial precancerous lesions and EC, potentially enhancing both the sensitivity and specificity of diagnosis.

### 2.2. lncRNAs in Cancer Metabolic Reprogramming: Linking Metabolites to Tumor Progression and Therapeutic Response

Cancer cells undergo extensive metabolic reprogramming to support rapid proliferation, survival, and adaptation to stress. Recent studies suggest that lncRNAs play a crucial role as key modulators of these metabolic pathways, linking specific metabolite alterations to tumor progression and treatment response. For instance, Shekher et al. [107] analyzed serum metabolomics profiles of breast cancer patients and matched healthy controls, identifying over 25 metabolites with altered levels, including elevated lactate, glycerol, and lactate/pyruvate ratio, along with decreased glucose, succinate, and isobutyrate. These changes reflect enhanced glycolysis and an altered lipid metabolism, hallmark features of cancer cells, and correlate with disease severity. These metabolic alterations are associated with the expression of several lncRNAs, including *H19*, *MEG3*, and *GAS5*. *H19* expression is upregulated in tumors compared to normal tissue, particularly in advanced-stage patients with lymph node involvement and after chemotherapy, suggesting its role in promoting tumor growth, modulating glucose metabolism, and contributing to chemoresistance. *MEG3*, although traditionally considered a tumor suppressor, is paradoxically elevated in certain advanced tumors, indicating condition-specific roles in disease progression. *GAS5* acts as a tumor suppressor with antiproliferative effects; its expression is increased in tumors, especially in advanced stages, and decreases after chemotherapy, making it a potential biomarker for tumor stage and therapeutic response.

In particular, *H19* and *MEG3* regulate metabolic pathways in gastric cancer. *H19* is associated with glucose metabolism and proliferation, while *MEG3* inhibits tumor growth and metastasis via the p53 signaling pathway. LncRNA-mediated metabolic regulation can even define tumor subtypes. Li and Ma [108] identified two gastric cancer subtypes, C1 and C2, with distinct metabolic and immune profiles, p53 mutation status, and chemotherapy sensitivities, demonstrating that lncRNAs integrate metabolic, genomic, and therapeutic features for patient stratification.

Finally, pathway analysis indicates that lncRNAs broadly regulate metabolism, with retinol, pyrimidine, purine, fatty acid, and arginine/proline metabolism being the most frequently affected [109]. These findings highlight the role of lncRNAs in regulating cancer metabolism and in linking metabolic alterations to tumor behavior, aggressiveness, and therapeutic response.

### 2.3. lncRNA and circRNA-Mediated Control of Cancer Energy Metabolism

Metabolic reprogramming is characterized by coordinated changes in carbohydrate, lipid, and amino acid metabolism that predominantly impact glycolysis, glutaminolysis, lipid metabolic pathways, and mitochondrial function [110,111]. LncRNAs have been identified as important regulators of these processes [112,113,114,115,116,117] and, in cancer, aberrant lncRNAs acting as oncogenes or tumor suppressors correlate with metabolic alterations [118,119].

A key feature of tumor metabolism is the Warburg effect, whereby cancer cells rely on glycolysis even in the presence of oxygen [120]. This adaptation promotes survival under conditions of nutrient and oxygen deprivation within the tumor microenvironment and supports rapid proliferation by supplying biosynthetic precursors and preserving redox homeostasis [110,121,122,123,124]. While most studies focus on protein-coding genes, ncRNAs play critical regulatory roles in these metabolic changes [125], modulating them through multiple mechanisms. In this context, their relationship with HIF-1α and c-Myc emerges as pivotal, and independently or cooperatively, they enhance glycolysis, promoting the Warburg effect [126,127,128,129,130,131,132]. An illustrative example is lncRNA *IDH1-AS1*, an antisense transcript of the *IDH1* gene transcribed from the opposite strand of the same genomic locus. Isocitrate dehydrogenase 1 (IDH1) and IDH2 are key enzymes in the Krebs cycle that regulate α-ketoglutarate levels and, indirectly, HIF-1α stability. Loss- or gain-of-function mutations in these enzymes alter TCA cycle activity, thereby promoting a metabolic reprogramming characterized by increased glycolytic dependence [133,134]. Although lncRNA *IDH1-AS1* does not directly regulate *IDH1* mRNA or protein levels, it enhances IDH1 enzymatic activity by promoting its homodimerization, thereby increasing α-KG production, reducing ROS, and subsequently promoting the proteasomal degradation of HIF-1α (via α-KG and prolyl hydroxylase), and suppressing the Warburg effect [135,136].

Regarding its interaction with c-Myc, *lncRNA-MIF* (c-Myc inhibitory factor) regulates cancer metabolism through a multilayered mechanism [137]. *LncRNA-MIF* is transcriptionally induced by c-Myc, establishing a negative feedback loop that restrains c-Myc activity. Mechanistically, *lncRNA-MIF* exerts its function as a ceRNA by sponging miR-586, which alleviates miRNA-mediated suppression of *FBXW7* and promotes its expression. Elevated FBXW7 promotes SCF–FBXW7-mediated ubiquitin–proteasome degradation of c-Myc, reducing its stability and transcriptional output. Through this coordinated control, *lncRNA-MIF* fine-tunes glycolytic signaling in cancer cells. Functionally, *lncRNA-MIF* overexpression reduces glucose uptake, lactate production, and the expression of key glycolytic genes, including *GLUT1*, whereas *lncRNA-MIF* silencing leads to c-Myc stabilization and enhanced aerobic glycolysis. Consistent with this role, *lncRNA-MIF* exhibits tumor-suppressive activity in multiple types of cancers by modulating the miR-586–FBXW7–c-Myc axis. In addition, in colorectal cancer, *circ-FBXW7* similarly displays anti-tumor effects by acting on the same pathway [138]. Given that aberrant c-Myc activation is a hallmark of many human cancers, the *lncRNA-MIF*–c-Myc feedback loop may represent a broader mechanism through which lncRNAs contribute to metabolic adaptation and tumor progression.

*LncRNA-MIF* exemplifies how a single lncRNA can integrate transcriptional regulation, miRNA interaction, and protein turnover to fine-tune cancer energy metabolism and support metabolic adaptation under oncogenic stress [137,139,140].

### 2.4. Involvement of lncRNAs and circRNAs in Glucose Metabolism

#### 2.4.1. lncRNAs in Glucose Metabolism

Altered glucose metabolism is a hallmark of cancer, as glucose serves as the primary carbon source for both cellular biosynthesis and energy production [120]. In colorectal cancer, ncRNAs are increasingly recognized as key regulators of glucose metabolism, with lncRNAs representing the most extensively investigated subclass. Available evidence suggests that lncRNAs influence key steps of glycolysis by modulating glucose transporters, glycolytic enzymes, and oncogenic signaling pathways, for example, in colorectal cancer [141]. Through these regulatory mechanisms, lncRNAs contribute to metabolic adaptation under conditions of nutrient fluctuation and hypoxia, supporting tumor cell survival and progression. In this context, lncRNAs frequently act as molecular scaffolds or transcriptional regulators that link metabolic cues to oncogenic programs, thereby contributing to metabolic reprogramming. Transcription factors dynamically modulate lncRNA expression in response to nutrient availability, thereby shaping metabolic adaptation. Under glucose restriction, the tumor suppressor p53, frequently mutated or deleted in many cancers, plays a central role in metabolic regulation by influencing glycolysis, oxidative phosphorylation, and the pentose phosphate pathway [142]. In this context, p53 transcriptionally activates specific lncRNAs that participate in the cellular response to metabolic stress. One example is *TRINGS* (*Tp53-regulated inhibitor of necrosis*), which inhibits the STRAP–GSK3β–NF-κB necrotic pathway and supports tumor cell survival under low-glucose conditions, with functional validation in vitro and in vivo [143]. Another p53-responsive lncRNA, *lincRNA-p21*, contributes to metabolic regulation by modulation of the PTEN/AKT/mTOR axis and HIF-1α signaling. In prostate cancer, downregulation of *lincRNA-p21* is associated with activation of the PTEN/AKT/mTOR pathway and upregulation of glycolytic enzymes, favoring tumor progression. Conversely, under hypoxic conditions, *lincRNA-p21* can be transiently induced by HIF-1α and bind to it, preventing its degradation by disrupting the HIF-1α–VHL interaction and sustaining glycolytic flux [144]. In parallel with stress-responsive pathways, oncogenic drivers, such as c-Myc, directly regulate glycolytic genes, reinforcing metabolic reprogramming [4]. Nutrient availability, particularly serine, further modulates glycolytic control through pyruvate kinase, the enzyme catalyzing the final and rate-limiting step of glycolysis. The *PKM* gene generates two alternatively spliced isoforms, PKM1 (Pyruvate Kinase, Muscle type, isoform 1) and PKM2. Whereas PKM1 is constitutively active and supports efficient ATP production in differentiated tissues, PKM2 predominates in proliferating and cancer cells and can exist in a highly active tetrameric form or a less active dimeric form. The dimeric configuration slows pyruvate generation, allowing upstream glycolytic intermediates to be diverted toward anabolic pathways that sustain tumor growth. PKM2 activity is also regulated allosterically. Indeed, serine binding promotes tetramer formation and enhances enzymatic activity, whereas low levels of serine decrease PKM2 activity, limiting pyruvate and ATP production. Under these conditions, cancer cells can compensate by activating *de novo* serine biosynthesis from the glycolytic intermediate 3-phosphoglycerate [145], illustrating the tight coupling between nutrient sensing and glycolytic flux. In this context, the c-Myc-responsive lncRNA *glycoLINC* (*gLINC*) acts as a scaffold that promotes assembly of a multi-enzyme complex comprising LDHA, PGK1, PGAM1, ENO1, and PKM2, thereby sustaining glycolytic flux and ATP production and supporting cancer cell survival under serine deprivation [146]. Consistently, *gLINC* silencing reduces tumor growth in xenograft models under serine-free conditions, highlighting its role in metabolic adaptation [146]. Complementary regulation of PKM2 can also occur at the level of splicing. For example, the hypoxia-induced circRNA *hsa_circ_0065394* encodes the peptide cPFKFB4 in pancreatic cancer, which promotes the alternative splicing switch from *PKM1* to *PKM2* by interacting with hnRNP G and interfering with its association with the splicing factor hnRNP A1, thereby reinforcing PKM2-dependent glycolytic reprogramming and sustaining the Warburg phenotype [68].

**Figure 1:**
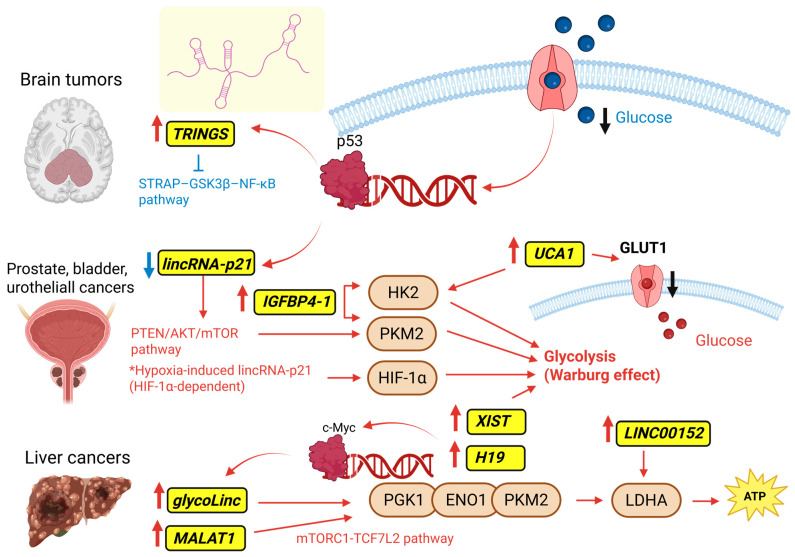
LncRNA-mediated regulation of cancer glucose metabolism. The schematic diagram shows representative mechanisms by which lncRNAs regulate metabolic pathways in different tumor settings. Here are reported the lncRNAs which have been shown to be most directly related to glycolysis/Warburg effects. Red arrows indicate an increase, whereas Blue (or Black) arrows indicate a decrease. LncRNAs are highlighted in yellow. Created with BioRender.com.

In summary, the interaction among transcription factors, lncRNAs, and glycolytic enzymes forms a regulatory network that enables cancer cells to adapt glucose metabolism to environmental and nutrient stress, supporting tumor growth. The schema in Figure 1 describes how distinct lncRNAs, acting in a tumor- and context-dependent manner, modulate key regulators of glycolysis through transcriptional control, signaling pathway modulation, and direct interaction with glycolytic enzymes. By integrating upstream transcription factors, such as p53 and c-Myc, with downstream metabolic targets including glucose transporters and glycolytic enzymes, the figure provides a mechanistic framework illustrating how lncRNAs contribute to metabolic adaptation, tumor progression, and survival under nutrient and hypoxic stress.

#### 2.4.2. circRNAs in Glucose Metabolism

In addition to lncRNAs, circRNAs have emerged as important regulators of the Warburg effect; however, mechanistic studies remain relatively limited, as seen in colorectal cancer [141]. Available evidence suggests that circRNAs contribute to glycolytic control primarily through miRNA sponging or interaction with metabolic regulators, thereby influencing glucose uptake and the activity of glycolytic enzymes. Rather than acting through isolated targets, most described circRNAs converge on key metabolic transcriptional hubs, such as HIF-1α and c-Myc, reinforcing glycolytic reprogramming under stress conditions.

CircRNAs can modulate glycolysis by interacting with miRNAs or proteins, often in response to hypoxic or metabolic stress. For example, in liver cancer cells, hypoxia-induced *circMAT2B* acts as a sponge for miR-338-3p, relieving its repression of PKM2 and thereby promoting glycolytic flux [147]. Similarly, in breast cancer cells, *circRNF20* sequesters miR-487a, which derepresses HIF-1α, leading to the upregulation of Hexokinase 2 (HK2) and enhanced glycolysis under hypoxic conditions [148]. These examples illustrate a recurring regulatory logic in which circRNAs amplify hypoxia-responsive glycolytic programs by relieving repression of central metabolic drivers rather than directly targeting single glycolytic enzymes. In line with these observations, growing evidence in breast cancer indicates that circRNAs play a pivotal role in glucose metabolic reprogramming, thereby influencing tumor behaviors. These findings highlight circRNAs as key contributors to metabolic plasticity, especially in breast cancer, suggesting their potential value as therapeutic targets [149].

Related to aerobic glycolysis, *circSLIT2* has been identified as upregulated and of prognostic relevance in gastric cancer. In pancreatic ductal adenocarcinoma (PDAC), *circSLIT2* was shown to act as a cytoplasmic miRNA sponge for miR-510-5p, targeting the 3′-UTR of c-Myc. In this manner, *circSLIT2* derepresses *c-Myc* [150], which in turn drives LDHA transcription and promotes glucose uptake and lactate production (Warburg phenotype). As these observations derive from different tumor types, we note that the miR-510-5p/c-Myc/LDHA axis has been mechanistically validated in PDAC but not yet in gastric cancer [151]. This distinction highlights the tumor context dependency of circRNA-mediated metabolic axes, even when similar regulatory architectures are observed. CircRNAs can enhance c-Myc functions through various mechanisms, including protein interactions that stabilize its protein, facilitate nuclear localization, or increase its transcriptional activity [40]. Similarly, *circSLC25A16* sponges miR-488-3p, thereby relieving HIF-1α repression in non-small cell lung cancer (NSCLC). The increased HIF-1α levels enhance LDHA transcription, further promoting glycolysis. In the same tumor context, *hsa_circ_103089* has been shown to enhance aerobic glycolysis and malignant progression by acting as a sponge for miR-876-5p, thereby upregulating *EGFR* expression [152]. Activation of the miR-876-5p/EGFR axis increases glycolytic activity, supports NSCLC cell migration and invasion, and reduces sensitivity to cisplatin treatment. These findings reinforce the concept that circRNAs integrate oncogenic signaling with metabolic reprogramming rather than acting as redundant regulators of individual glycolytic enzymes.

Conversely, tumor-suppressive circRNAs limit glycolysis by inhibiting key metabolic regulators. *CircNR3C1*, for instance, prevents c-Myc from forming a transcriptional complex with BRD4, reducing c-Myc target gene expression in bladder cancer [58]. Similarly, in gastric cancer, *circSCMH1* acts as a tumor-suppressive circRNA that inhibits aerobic glycolysis and metastatic potential by sponging miR-296–3p [153]. Through the miR-296–3p/HSPB7–GLUT3 axis, *circSCMH1* reduces glucose uptake and glycolytic flux, thereby limiting energy availability required for tumor cell migration and invasion. *CircRNF13*, which is downregulated in nasopharyngeal carcinoma (NPC), stabilizes *SUMO2* mRNA, leading to GLUT1 degradation and subsequent inhibition of glycolysis [154]. *CircRPN2* in HCC exerts dual tumor-suppressive functions by binding ENO1 to promote ubiquitin-mediated degradation and by sponging miR-183-5p, which normally represses *FOXO1*, thereby suppressing glycolysis and tumor progression [155]. These suppressive mechanisms underscore the ability of circRNAs to counteract metabolic activation, either by promoting the degradation of glycolytic enzymes or by reinstating tumor-suppressive transcriptional programs.

Beyond mRNA-based mechanisms, circRNAs can also exert effects through peptide expression or protein stabilization. *CircFNDC3B*, for instance, encodes a 218-amino-acid peptide (circFNDC3B-218aa) that inhibits the EMT regulator Snail, indirectly promoting the expression of Fructose-1,6-bisphosphatase 1, a key enzyme in glucose metabolism, thereby modulating the balance between glycolysis and gluconeogenesis in colon cancer cells [156]. This example expands the regulatory spectrum of circRNAs beyond ceRNA activity, emphasizing their potential coding capacity in metabolic control.

Another prominent example is *circACC1*, derived from exons 2–4 of the pre-mRNA of Acetyl-CoA Carboxylase 1 (ACC1), which functions as a metabolic stress sensor in colorectal cancer. Under conditions of serum deprivation, *circACC1* is upregulated through activation of the JNK/c-Jun pathway, which promotes its circularization at the expense of linear *ACC1* mRNA production. *CircACC1* binds specifically to the β1 and γ1 subunits of AMPK, stabilizing the complex, preventing ubiquitination and degradation, and enhancing kinase activity. This RNA-mediated stabilization mimics allosteric activation of AMPK, promoting glycolysis, β-oxidation, and ATP/NADPH homeostasis, while reducing lipid accumulation [157]. Consequently, *circACC1* supports cancer cell survival under nutrient-limited conditions, and its overexpression enhances tumor growth, whereas knockdown reduces aggressive behavior in colorectal cancer cells [158,159].

**Figure 2:**
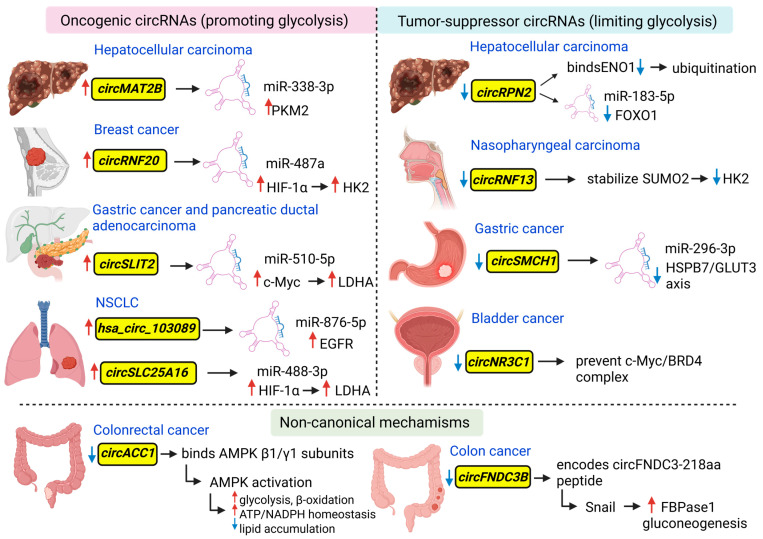
CircRNA-mediated regulation of glucose metabolism in cancer. This schema summarizes representative oncogenic and tumor-suppressive roles of circRNAs in the regulation of glucose metabolism across different cancer types. Here, we illustrate the circRNAs for which a well-defined molecular axis has been described, and a clear functional mechanism has been experimentally validated. Oncogenic circRNAs (**left panel**) promote glycolysis primarily through miRNA sponging mechanisms. In contrast, tumor-suppressive circRNAs (**right panel**) limit glycolysis by inhibiting key metabolic regulators. Red arrows indicate an increase, whereas blue arrows indicate a decrease. CircRNAs are highlighted in yellow. Figure created with BioRender.com.

Together, these examples (reported in Figure 2) demonstrate that circRNAs contribute to cancer metabolic reprogramming through diverse mechanisms, including miRNA sponging, peptide production, and direct protein interaction, highlighting their multifaceted roles in promoting glycolysis and supporting tumor progression. Overall, circRNAs emerge not merely as passive correlates of metabolic rewiring but as dynamic integrators of hypoxic signaling, oncogenic transcriptional programs, and nutrient stress adaptation.

### 2.5. mTOR–ncRNA Crosstalk in Cancer Cell Growth and Metabolism

The mammalian target of rapamycin (mTOR) is a central regulator of cell growth and metabolism [160]. In particular, mTOR complex 1 (mTORC1) is the molecular target of rapamycin and its analogs, which are employed in therapy for various carcinomas. The rapid proliferation and invasive potential of cancer cells are largely driven by activation of the mTORC1 pathway, which promotes the Warburg effect and shifts cellular metabolism toward aerobic glycolysis. mTORC1 also stimulates glycolysis indirectly through HIF-1α-dependent transcription of glycolytic enzymes under hypoxic conditions. Clinically, HCC frequently exhibits oncogenic activation of the mTOR signaling pathway. Experimental models, including genetically engineered mice and hydrodynamic transfection systems, have demonstrated that increased mTORC1 activity contributes to the initiation of hepatocarcinogenesis [161]. Despite this strong mechanistic rationale, recent clinical trials evaluating rapamycin analogs, including everolimus and temsirolimus, in patients with advanced HCC have not met significant therapeutic endpoints [162]. This underscores the need to explore combinatorial or alternative strategies to improve the clinical efficacy of mTOR-targeted therapies.

mTORC1 also regulates the lncRNA transcriptome. Notably, *NEAT1*, a key organizer of nuclear paraspeckles, is a downstream target of mTORC1. *NEAT1* forms paraspeckles by polymerizing with RBPs, such as NONO and SFPQ [163]. Interestingly, HCC tumors exhibit marked downregulation of *NEAT1*, particularly the *NEAT1_2* isoform, correlating with poor overall survival. Suppression of *NEAT1_2* by mTORC1 promotes mRNA splicing, the expression of glycolytic enzymes, and the Warburg effect, thereby linking nuclear architecture to cancer metabolism. Overall, mTORC1-driven modulation of *NEAT1_2* and paraspeckle biogenesis influences liver tumor development, metabolic reprogramming, and responsiveness to mTORC1-targeted therapies [164].

On the other hand, several circRNAs control the AKT/mTOR signaling pathway, which also regulates metabolism. For instance, *circPPP1R12A* is upregulated in NSCLC by activating this pathway, which promotes cell proliferation while reducing apoptosis [165]. Conversely, *circCGNL1* in NPC is downregulated because it exerts tumor-suppressive activity by sequestering Insulin-like growth factor 2 mRNA-binding protein 3 (IGF2BP3), which, when released, leads to AKT and mTOR phosphorylation, promoting proliferation while suppressing apoptosis [166]. In colon cancer, it was found that the peptide encoded by *circPPP1R12A* promoted the growth and metastasis by activating the Hippo-YAP signaling pathway [167].

Additional examples include circRNAs involved in the MAPK signaling/mTOR pathway. *CircZKSCAN1* drives lung adenocarcinoma progression and chemoresistance by sponging miR-185-5p, which targets *TAGLN2*, an actin-binding protein, indirectly enhancing glycolytic flux [168]. In NSCLC, this oncogenic circRNA has been shown to sponge miR-330-5p, thereby relieving its inhibitory effect on the expression of FAM83A, a protein involved in activation of the MAPK signaling pathway [169]. In HCC, *circZKSCAN1* is downregulated at baseline but re-induced by sorafenib via FBXW7 [170]. In this setting, its miRNA-sponging activity appears limited; instead, it encodes a tumor-suppressive peptide that promotes FBXW7-mediated mTOR degradation and inhibits oncogenic signaling.

**Figure 3:**
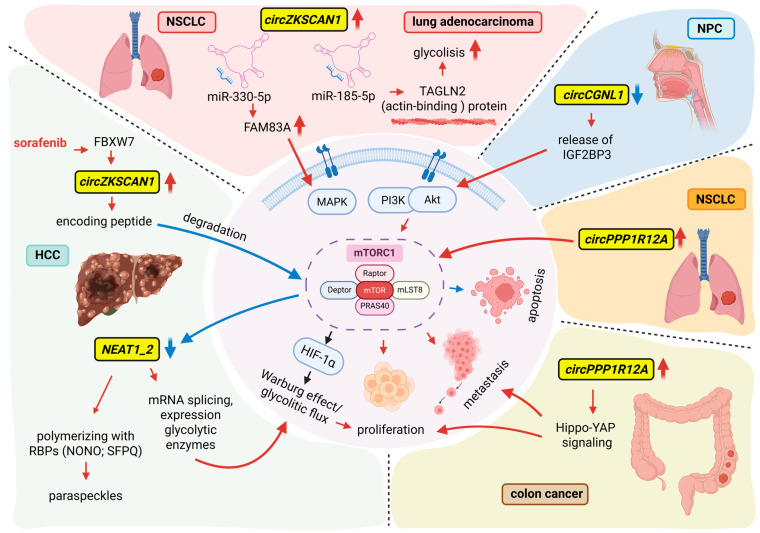
Crosstalk between mTORC1 signaling and non-coding RNAs in cancer growth and metabolism. This cartoon represents the main lncRNAs and circRNAs whose mechanisms of action have been experimentally validated that regulate, or are regulated by, the PI3K–Akt–mTOR and MAPK signaling pathways across different tumor types. Red arrows indicate activation or upregulation of signaling pathways, molecular interactions, or biological processes, whereas Blue arrows denote inhibitory effects, downregulation, or degradation events. Yellow boxes highlight circRNAs whose expression is altered in specific cancers, with upward or downward arrows indicating increased or decreased expression, respectively. Figure created with BioRender.com.

Figure 3 summarizes representative mechanisms by which lncRNAs and circRNAs modulate mTOR signaling and cancer-associated metabolic and survival pathways in distinct tumor contexts.

### 2.6. lncRNAs and circRNAs in Glutamine Metabolism

To sustain the high biosynthetic and energetic demands of proliferation, cancer cells must significantly increase nutrient uptake from the extracellular environment. In addition to glucose, glutamine is particularly critical, as its catabolism provides carbon intermediates for macromolecule synthesis and reducing equivalents (NADH, FADH2) necessary for ATP production, redox homeostasis, and biosynthetic processes (NADPH) [171,172]. While glucose fuels glycolysis and the Warburg effect, glutamine serves as both a carbon and nitrogen donor, supporting the synthesis of nucleotides, glucosamine-6-phosphate, and non-essential amino acids. Intracellular glutamine also promotes the uptake of essential amino acids through LAT-mediated exchange mechanisms [173].

Glutamine metabolism is tightly regulated by oncogenic signaling pathways. For instance, c-Myc overexpression in proliferative and cancer cells enhances glutamine uptake by inducing the expression of glutamine transporters (ASCT2, SN2) and key metabolizing enzymes (GLS1, PRPS2, CAD) [174]. This increases the production of glutamate, linking glutamine catabolism to the Krebs cycle anaplerosis, redox regulation, and cystine uptake via xCT, thereby maintaining cellular homeostasis under proliferative stress [175]. Additionally, the PI3K/Akt pathway, activated downstream of receptor tyrosine kinases and extracellular matrix signals, cooperates with glutamine metabolism by promoting glycolysis through upregulation of hexokinase, phosphofructokinase, and GLUT1 [176].

*Circ_0000517*, overexpressed in NSCLC, represents an example. It targets miR-330-5p, a negative regulator of the transcription factor YY1, resulting in increased glucose uptake, lactate production, ATP generation, glutamine consumption, glutamate content, α-ketoglutarate levels, and cell proliferation [177]. In general, multiple circRNAs across different types of cancer sponge miRNAs regulating SLC family transporters responsible for glutamine uptake, highlighting their role in coordinating both glucose and amino acid metabolism [178]. In addition, *circular Membrane Bound O-Acyltransferase Domain Containing 2* (*circ-MBOAT2*) upregulated in pancreatic cancer sponges miR-433-3p, which associates with the 3′UTR of glutamic-oxaloacetic transaminase 1 mRNA, an enzyme necessary for cancer to provide amino acids [179]. Similarly, *circular RNA CREB-binding protein* (*circ-CREBBP*) is highly expressed in glioma and upregulates glutaminase expression by sponging miR-375 [180].

Several types of cancers exhibit a pronounced dependence on glutamine. For example, triple-negative breast cancer (TNBC) shows reduced glutamine levels, elevated glutamate concentrations, and increased reliance on glutamine compared with non-TNBC tumors, reflecting this metabolic rewiring [181]. When glutamine becomes limiting, adaptive stress responses are triggered. A notable example involves the lncRNA *MLLT4-AS1*, which encodes the 21-amino-acid micropeptide XBP1SBM. Under glutamine starvation conditions, activation of the IRE1α–XBP1s axis induces *MLLT4-AS1*/XBP1SBM, which binds to XBP1s, retains it in the nucleus, and enhances its occupancy at the VEGF promoter. This mechanism promotes angiogenesis and tumor aggressiveness, as validated in vivo. Indeed, suppression of XBP1SBM translation or deletion of its coding sequence reduces both angiogenesis and tumor growth [182].

**Figure 4:**
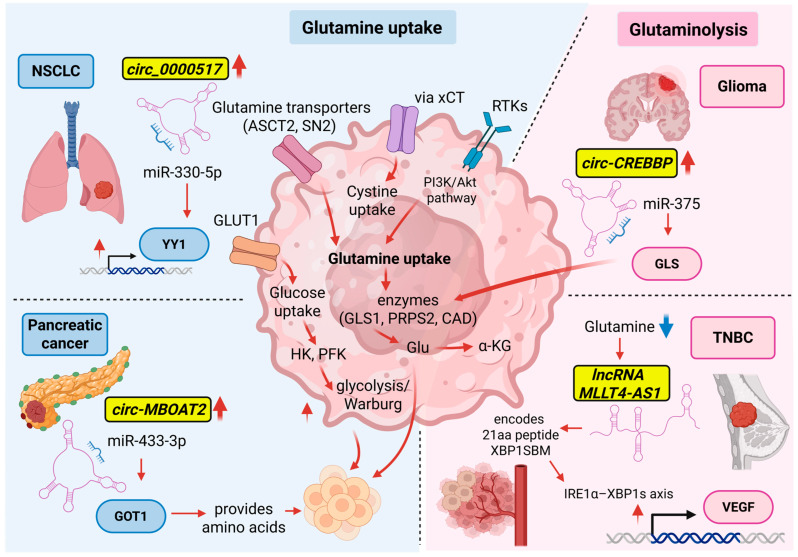
NcRNA-mediated regulation of glutamine uptake and glutaminolysis in cancer. This schematic summarizes representative mechanisms by which circRNAs and lncRNAs modulate glutamine uptake and glutaminolysis across different cancer types, including NSCLC, pancreatic cancer, glioma, and TNBC. Red arrows indicate activation or upregulation of molecular pathways, metabolic processes, or gene expression, whereas Blue arrows denote inhibition or downregulation. Black arrow indicates gene expression. Alterations in ncRNA expression affect glutamine transporters, key metabolic enzymes, and downstream signaling pathways, ultimately supporting metabolic reprogramming, proliferation, and tumor progression. NcRNAs are highlighted in yellow. Figure created with BioRender.com.

These findings, summarized in Figure 4, emphasize the crucial role of glutamine in cancer metabolism and demonstrate how these ncRNAs, through mechanisms such as peptide encoding, transcriptional regulation, or protein interaction, act as key modulators of nutrient utilization, metabolic adaptation, and tumor progression.

### 2.7. Lipid Metabolism and lncRNA Regulation in Cancer

In many cancers, lipid metabolism is reprogrammed to enhance *de novo* lipogenesis while dynamically integrating exogenous lipid uptake, enabling metabolic flexibility under diverse microenvironmental conditions. Acetyl-CoA is first carboxylated to malonyl-CoA by ACC1/2 and then condensed by FASN to generate palmitate (C16:0). Palmitate can be further elongated or desaturated by enzymes such as ELOVLs, SCD/SCD5, and FADS1-3, producing longer or unsaturated fatty acids necessary for membrane synthesis and energy storage. Lipogenic reprogramming is tightly controlled by oncogenic signaling pathways. The AMPK pathway, downstream of the tumor suppressor STK11/LKB1, phosphorylates ACC to inhibit fatty acid synthesis, acting as a metabolic checkpoint. Conversely, the PI3K/Akt axis stimulates lipid synthesis by upregulating lipogenic enzymes and activating ATP-citrate lyase (ACLY), thereby enhancing acetyl-CoA availability. As a consequence, cancer cells exhibit a lipid composition enriched in saturated and monounsaturated fatty acids. This adaptation decreases lipid peroxidation, shielding cells from oxidative damage and supporting tumor growth, metastasis, and survival under nutrient stress.

Within this framework, lncRNAs emerge as critical modulators, linking oncogenic signaling to metabolic outcomes. *Linc-ADAL* coordinates the expression of key lipogenic genes, including *ACLY*, *ACC*, and *FASN*, by interacting with hnRNPU to promote transcriptional activation and with IGF2BP2 to enhance mRNA stability and translation [183]. In this way, *linc-ADAL* influences fatty acid production and adipocyte differentiation. Other lncRNAs function as bona fide oncogenic, such as *NEAT1*, which promotes PPARα-dependent fatty acid oxidation and sustains ATGL-mediated lipolysis [184], facilitating HCC growth and forming a feedback loop with *TP53* in a context-dependent manner, while *Highly Upregulated in Liver cancer* (*HUCL*) increases *ACSL1* and genes involved in fatty acid activation/oxidation by relieving miR-9-mediated repression of PPARα [185]. In cervical cancer, a lncRNA associated with lymph node metastasis in cervical cancer (*LNMICC*) recruits Nucleophosmin 1 (NPM1) to the promoter of Fatty Acid Binding Protein 5 [186], enhancing fatty acid metabolism and promoting metastasis. Interestingly, not all lncRNAs support uniformly tumor progression leading to β-oxidation. For example, *lncHR1* represents a tumor-suppressive lncRNA that suppresses SREBP-1c and FASN [187], reducing triglyceride accumulation in HCC.

In esophageal squamous cell carcinoma (ESCC), palmitic acid and high-fat diets inhibit tumor growth, at least in part, by downregulating the oncogenic lncRNA *SLC25A21-AS1*, which is otherwise upregulated in ESCC. When expressed, *SLC25A21-AS1* stabilizes *SLC25A21* mRNA in the cytoplasm, modulating tryptophan catabolism, energy metabolism, and NAD^+^ biosynthesis, and interacts with NPM1 in the nucleus to promote c-Myc transcription [188]. Through these cytoplasmic and nuclear mechanisms, *SLC25A21-AS1* enhances tumor cell proliferation, migration, metastasis, and cisplatin resistance, whereas its downregulation abrogates these oncogenic effects, highlighting its potential as a prognostic biomarker and therapeutic target in ESCC.

**Figure 5:**
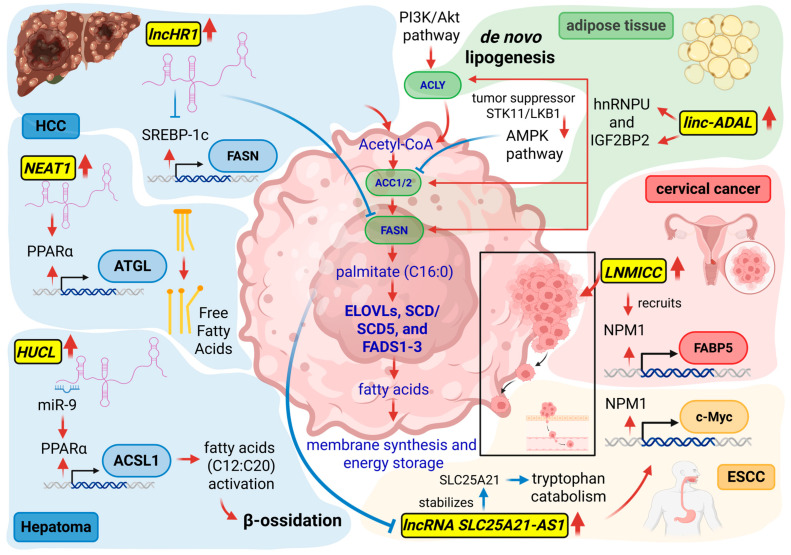
LncRNA-mediated regulation of lipid metabolism in cancer. Red arrows indicate activation or upregulation of metabolic pathways, enzymes, or gene expression, whereas blue bar-headed line denote inhibition or suppression of the indicated processes. Black arrow indicates gene expression. Central carbon flux through *de novo* lipogenesis is illustrated, highlighting the conversion of citrate-derived acetyl-CoA by ACLY and ACC1/2, followed by fatty acid synthesis mediated by FASN and subsequent elongation and desaturation via ELOVLs, SCD/SCD5, and FADS1–3, thereby supporting membrane biosynthesis and energy storage. LncRNAs highlighted in yellow modulate lipid metabolism in a cancer-type-specific manner. Figure created with BioRender.com.

These lncRNAs were depicted in Figure 5, as well as their mechanisms of action and cancer specificity.

## 3. An Attractive Prospect: *OTX2-AS1*, Indirect Metabolic Influence and Predictive Biomarker in Medulloblastoma

*Orthodenticle Homeobox 2 Antisense RNA 1* (*OTX2-AS1*) is a lncRNA transcribed from the proximal promoter of the transcription factor OTX2 in the opposite direction. Promoter hypermethylation in the Sonic Hedgehog subgroup reduces its expression, mirroring the pattern of OTX2 itself. *OTX2-AS1* has not been directly linked to canonical metabolic enzymes or pathways. Proteomic analysis following its knockout revealed upregulation of mitochondrial oxidative phosphorylation proteins, including succinate dehydrogenase subunit B, suggesting an indirect role in modulating the balance between glycolytic and oxidative metabolism in tumor cells. These observations imply that *OTX2-AS1* may contribute to the metabolic plasticity of medulloblastoma, favoring proliferation and survival in glycolysis-dependent states.

In medulloblastoma, *OTX2-AS1* is highly expressed in the WNT signaling pathway, Group 3, and Group 4 molecular subtypes, and its elevated levels correlate with increased sensitivity to BCL-2 inhibitors, as demonstrated by drug screening assays. These findings suggest that patients with high *OTX2-AS1* expression may benefit from combination therapy involving conventional treatments and BCL-2-targeted agents. Accordingly, *OTX2-AS1* may serve both as an indicator of tumor subtype and a predictor of therapeutic response, highlighting its dual potential in clinical stratification and treatment optimization [189].

## 4. ncRNA-Based Therapies

NcRNAs play a central role in cancer, and their tissue specificity, dynamic expression, and stability further support their value as biomarkers. Clinical translation is already underway. Ongoing trials, including the EOC-EXOSOME study (NCT03738319), are evaluating exosomal miRNA and lncRNA signatures as diagnostic and prognostic tools [190].

On the other hand, they may serve as candidates for therapeutic targeting. Moreover, their dysregulation may provide opportunities for innovative therapeutic strategies, including exosome-based delivery systems and CRISPR-mediated interventions [191].

Currently, in gastric cancer, lncRNAs have emerged as major regulators of disease progression and treatment response through diverse mechanisms affecting proliferation, EMT, and metabolic adaptation [192]. Many lncRNAs exert their oncogenic or tumor-suppressive functions through miRNA-mediated post-transcriptional regulation. For example, *XIST* functions as a sponge for miR-149-3p, influencing tumor growth and metastasis; its downregulation in OC correlates with shorter progression-free survival, BRCA1 mutations, enhanced proliferation and invasion, and resistance to cisplatin and paclitaxel [193]. Similarly, *PVT1* promotes disease progression by recruiting EZH2 to repress miR-214 transcription, and its amplification in advanced OC is associated with poor prognosis and patient risk stratification [194]. Other lncRNAs, including *HOTAIR*, *ANRIL*, and *SNHG16*, contribute to metastasis and therapy resistance across many types of cancers, highlighting the importance of ncRNA-mediated transcriptional and post-transcriptional regulation in tumor evolution [195,196,197].

Also, circRNAs contribute to the pathogenesis of gastric and ovarian cancers through mechanisms including miRNA sponging, protein interactions, and, in some cases, peptide encoding. By modulating oncogenic signaling and stress-adaptive responses, aberrant expression of circRNAs promotes tumor progression and therapeutic resistance. Their high stability and detectability in tissues and biofluids make them attractive non-invasive biomarkers and potential therapeutic targets. For instance, *circHIPK3* exerts tumor-suppressive effects in OC [198], whereas other circRNAs have been linked to chemotherapy resistance and aggressive phenotypes.

A pathway-oriented reorganization of the lncRNAs and circRNAs identified in this study is presented in Table 1, highlighting the enrichment of specific tumor types in ncRNAs implicated in metabolic reprogramming. In particular, HCC, colorectal cancer, and gastric cancer show the greatest enrichment of metabolism-related lncRNAs and circRNAs, encompassing pathways involved in glycolysis, glutaminolysis, lipid metabolism, redox homeostasis, and nutrient sensing. This recurrent identification of multiple panels of regulators within the same tumor context suggests that metabolic plasticity in these malignancies is sustained by multilayered non-coding regulatory networks rather than by isolated molecular events. From a translational perspective, such enrichment indicates that these tumor types may already harbor a repertoire of ncRNA candidates with therapeutic potential, either as direct metabolic targets or as modulators of key oncogenic hubs such as c-Myc, HIF-1α, AMPK, and mTOR. Conversely, other tumor entities remain comparatively underrepresented, highlighting potential gaps in current research rather than a true absence of ncRNA-mediated metabolic control.

Overall, these observations support the concept that tumor-specific ncRNA landscapes may help prioritize metabolism-oriented therapeutic strategies and guide the development of ncRNA-informed precision oncology approaches.

## 5. Limitations of the Current Evidence and Future Research Directions in Linking ncRNAs to Cancer Metabolism

Despite the rapidly expanding literature implicating lncRNAs and circRNAs in cancer metabolic reprogramming, several conceptual, experimental, and technical limitations currently constrain the strength, reproducibility, and translational relevance of these findings. A central issue that permeates much of the field is the frequent lack of distinction between correlation and causality in reported ncRNA–metabolism relationships. In many studies, metabolic regulation is inferred from concordant changes in ncRNA expression and metabolic gene signatures, pathway enrichment analyses, or steady-state metabolite levels. However, given that cellular metabolism is governed by multiple regulatory layers (including enzyme kinetics, substrate availability, compartmentalization, and allosteric control), such associations are insufficient to demonstrate direct functional regulation of metabolic pathways [199]. This limitation applies to both lncRNAs and circRNAs, particularly when functional validation relies primarily on proliferation assays, survival phenotypes, or transcriptional changes rather than on direct measurements of metabolic activity.

This difficulty in establishing causality is closely linked to another major limitation of the current literature, namely, the limited integration of ncRNA studies with quantitative and dynamic measurements of metabolism. Most investigations still rely on indirect readouts, such as glucose uptake, lactate secretion, or expression of metabolic enzymes, which do not capture pathway flux or metabolic plasticity. Stable isotope tracing combined with mass spectrometry or NMR spectroscopy represents the gold standard for quantifying metabolic fluxes yet remains underutilized in lncRNA- and circRNA-focused studies [200]. Emerging approaches (including high-resolution fluxomics, genetically encoded biosensors for ATP, NADH/NAD^+^, and redox state, as well as dynamic nuclear polarization-enhanced MRI) offer new opportunities to directly assess metabolic activity downstream of ncRNA perturbation. In parallel, spatial metabolomics and mass spectrometry imaging (MSI) technologies have emerged as powerful tools for region-specific metabolic profiling directly in tissue sections, preserving spatial information that is lost in bulk metabolomic analyses. Techniques such as matrix-assisted laser desorption/ionization (MALDI)–MSI and desorption electrospray ionization (DESI)–MSI enable the visualization of metabolites, lipids, and small molecules across histological contexts, thereby revealing intratumoral metabolic heterogeneity and metabolic gradients associated with tumor architecture and microenvironmental niches [201]. These approaches are increasingly being applied in cancer research to map spatially resolved metabolic states associated with hypoxia, necrosis, invasive fronts, and therapeutic response.

Recent advances in spatial metabolomics, integrating MSI with advanced computational and analytical frameworks, have enhanced sensitivity, expanded molecular coverage, and improved spatial resolution, thereby reinforcing the potential of these technologies for integrative multi-omics applications [202,203]. Notably, while direct integration of spatial metabolomics with spatial transcriptomics remains an emerging frontier, several recent studies have begun to combine spatial metabolic profiling with transcriptomic data to reveal regionally heterogeneous metabolism in intact tissues. For example, combining spatial metabolomics with transcriptome profiling has uncovered metabolic heterogeneity and differential gene–metabolite associations in disease models [204]. Comprehensive reviews of spatial multi-omics also highlight ongoing efforts to align diverse spatial measurements, laying a methodological foundation for future ncRNA-centric analyses [205]. Nevertheless, formal integration of spatially resolved ncRNA expression, including lncRNAs and circRNAs, with spatial metabolite profiles remains largely unexplored, representing a key opportunity for future research.

Beyond measurement-related limitations, biological context represents an additional and often underappreciated source of variability. Metabolic programs differ markedly according to tissue lineage, oncogenic driver mutations, oxygen and nutrient availability, immune interactions, and therapeutic pressure. Nevertheless, many ncRNA–metabolism links have been reported in a narrow range of experimental models, often restricted to one or two tumor types or even single cell lines. This limitation is further exacerbated by the extensive use of long-established cancer cell lines, which frequently harbor profoundly abnormal genomes characterized by aneuploidy, large chromosomal deletions or amplifications, and extensive karyotypic rearrangements. Such genomic distortions can profoundly reshape both transcriptional regulation and metabolic wiring, potentially generating ncRNA–metabolism relationships that are not representative of primary tumors in vivo [206].

The constraints imposed by experimental context are further amplified by the culture conditions under which most studies are performed. The majority of ncRNA metabolism investigations are conducted under normoxic, high-glucose, two-dimensional culture conditions, which poorly recapitulate the metabolic constraints of the tumor microenvironment. Oxygen gradients, nutrient deprivation, acidosis, stromal interactions, and therapy-induced stress are central determinants of tumor metabolism yet are rarely incorporated into mechanistic studies of lncRNAs or circRNAs. Hypoxia profoundly alters both ncRNA expression and metabolic flux, as HIFs coordinate extensive transcriptional and post-transcriptional programs under oxygen deprivation. Comprehensive analyses indicate that HIF activation not only promotes metabolic reprogramming but also modulates a broad spectrum of ncRNAs, which in turn contribute to hypoxic responses and metabolic adaptation [207]. Although more physiologically relevant systems (including organoids, spheroids, patient-derived cultures, and microfluidic platforms) offer improved modeling of metabolic stress, their adoption in ncRNA-focused metabolic studies remains limited.

In parallel with these biological and experimental challenges, intrinsic technical limitations complicate the study of ncRNA expression itself. LncRNAs are often expressed at low abundance, exhibit pronounced cell-type and cell-state specificity, and display complex subcellular localization patterns, complicating accurate quantification. CircRNAs introduce additional complexity due to their covalently closed structure, resistance to exonuclease degradation, and frequent overlap with linear host transcripts. Standard RNA sequencing approaches optimized for polyadenylated transcripts may therefore underrepresent or misclassify circRNAs, as these lack poly(A) tails and are frequently identified solely through back-splice junction reads, which are sensitive to library preparation and mapping artifacts, leading to incomplete or biased datasets [40,208,209]. Although methodological advances, including total RNA (rRNA-depleted) library preparation, RNase R-based enrichment, and the development of dedicated computational pipelines, have improved circRNA detection, considerable heterogeneity in experimental protocols and bioinformatic workflows persists, thereby limiting cross-study comparability and reproducibility [210,211].

Recent technological advances, particularly long-read sequencing approaches, are helping to overcome these limitations by enabling accurate reconstruction of full-length lncRNA and circRNA isoforms, while also resolving transcript structural complexity and back-splicing events that are difficult to capture with short-read sequencing [211,212]. Improved computational frameworks and curated resources now allow systematic analysis of ncRNA expression at single-cell resolution. For example, single-cell RNA sequencing studies have revealed that lncRNAs display pronounced cell-type- and cell-state-specific expression patterns, which are often masked in bulk transcriptomic analyses, leading to an underestimation of their biological relevance in heterogeneous tissues [213,214]. Dedicated resources such as LnCeCell 2.0 integrate single-cell and spatial transcriptomics data to map lncRNA-associated ceRNA networks and provide tools for exploring cell-specific regulatory relationships, although these platforms currently lack direct metabolic readouts [215].

CircRNAs have also begun to be explored at single-cell resolution, primarily through full-length single-cell RNA-seq approaches, which have revealed cell-type-specific and condition-dependent circRNA expression landscapes in both normal and tumor tissues [216]. While these studies establish the technical feasibility of detecting circRNAs at single-cell resolution, they remain largely disconnected from functional measurements of metabolism.

As a result, direct coupling of ncRNA expression with metabolic states at single-cell or spatial resolution remains limited. Although emerging single-cell metabolic inference methods and spatial transcriptomics platforms enable the indirect estimation of metabolic pathway activity, their integration with lncRNA or circRNA profiling in tumors remains limited. This gap is significant because ncRNAs are often cell-state-specific, while metabolic phenotypes vary across tumor regions in response to microenvironmental gradients.

Finally, these technical and experimental constraints converge on a broader conceptual challenge: reconciling the dynamic, spatially heterogeneous nature of tumor metabolism with the conditional and context-specific expression of ncRNAs. LncRNAs and circRNAs often function within narrowly defined transcriptional or stress-induced states, while metabolic programs evolve spatially and temporally during tumor progression and therapy. This mismatch suggests that many ncRNA–metabolism relationships may be transient, indirect, or condition-specific rather than stable regulatory axes.

These limitations underscore the need to move beyond predominantly correlative frameworks toward causal and quantitative strategies to study ncRNA-mediated metabolic regulation. Integrating advanced ncRNA detection with flux-based analyses, physiologically relevant models, and spatial or single-cell approaches will be essential to define their functional contribution to cancer metabolic plasticity and clinical translation. Taken together, these conceptual, technical, and contextual limitations provide an essential framework for interpreting the current literature and reinforce the need for a cautious and nuanced evaluation of ncRNA–metabolism relationships; in this context, the following discussion aims to integrate existing evidence, highlight biologically meaningful patterns, and delineate realistic opportunities for future investigation.

## 6. Discussion

Recent advances in cancer research underscore the pivotal role of lncRNAs and circRNAs in tumor metabolism and progression. At the same time, the evidence summarized in this review highlights that ncRNA-mediated metabolic regulation is highly context-specific and often inferred from correlative datasets, thereby emphasizing the importance of cautious interpretation. Both lncRNAs and circRNAs act as key modulators of glucose, lipid, and amino acid metabolism, frequently integrating oncogenic signaling pathways such as c-Myc, HIF-1α, and mTOR to sustain the metabolic demands of proliferating cancer cells.

Although lncRNAs such as *H19*, *MEG3*, and *HOTAIR* have been extensively studied in cancer biology, recent evidence indicates that their functions extend beyond canonical roles in signaling, epigenetic regulation, and EMT. In this study, these lncRNAs are specifically re-examined within a metabolic framework, highlighting their ability to modulate key metabolic pathways that support tumor progression, therapy resistance, and phenotypic plasticity. *H19* has been characterized as a regulator of aerobic glycolysis and glucose utilization, contributing to metabolic flexibility and chemoresistance in breast and gastric cancers by shaping glycolytic flux and lactate production, as supported by recent studies combining lncRNA profiling with metabolomic analysis. *MEG3*, traditionally regarded as a tumor suppressor, exhibits condition-specific metabolic functions and participates in p53-associated metabolic control, influencing glucose metabolism and mitochondrial activity, particularly in advanced disease settings. Similarly, *HOTAIR* links epigenetic remodeling to metabolic adaptation by coordinating chromatin-based regulation of genes involved in lipid metabolism and energy homeostasis. By integrating metabolic readouts, pathway-level analysis, and clinical correlations, this review demonstrates how these well-known lncRNAs act as dynamic regulators of cancer metabolism in a context- and stage-dependent manner, underscoring their relevance as biomarkers of metabolic vulnerability and potential targets for metabolism-oriented therapeutic strategies.

Similarly, *PVT1* is re-examined within a metabolic framework, and is positioned as a key lncRNA that links oncogenic signaling to metabolic reprogramming in cancer. Rather than being considered solely as a generic oncogenic transcript, *PVT1* is highlighted for its involvement in c-Myc-driven metabolic control, particularly affecting lipid metabolism in endometrial and other cancers, where its dysregulation correlates with tumor progression and aggressive metabolic phenotypes. Through its interaction with c-Myc and downstream transcriptional networks, *PVT1* contributes to the rewiring of lipid biosynthesis and energy metabolism. Evidence from studies integrating lncRNA expression with metabolomic profiling further indicates that *PVT1* expression is associated with coordinated alterations in lipid and amino acid metabolism. This integrated perspective positions *PVT1* as a dynamic metabolic hub that connects oncogenic cues, metabolite availability, and tumor progression, reinforcing its relevance as a biomarker of metabolic vulnerability and a potential target for metabolism-oriented therapeutic strategies.

Concerning circRNAs, *circACC1* and *circMAT2B* describe how circRNAs can regulate glycolysis, AMPK signaling, and metabolic stress adaptation through distinct molecular mechanisms, including miRNA-mediated derepression and direct stabilization of protein complexes. These observations support the view that ncRNAs contribute to metabolic rewiring rather than merely reflecting it.

To provide a clearer integrative overview and to reduce functional heterogeneity across examples, lncRNAs and circRNAs discussed in this review have been reorganized according to the major metabolic pathways they regulate (Table 2).

This pathway-oriented framework highlights common mechanistic themes across ncRNA classes. In the context of glycolysis and the Warburg effect, lncRNAs such as *H19*, *gLINC*, *MIF*, and *lincRNA-p21* promote glycolytic flux through miRNA sponging, enzyme scaffolding, or modulation of c-Myc and HIF-1α activity, whereas circRNAs, including *circMAT2B*, *circACC1*, *circSLIT2*, *circSLC25A16*, and *circRNF20*, enhance glucose uptake and ATP production through derepression of PKM2, stabilization of AMPK complexes, or activation of c-Myc-dependent transcriptional programs. Similarly, in glutaminolysis and amino acid metabolism, lncRNAs such as *MLLT4-AS1* and *LINC02313* regulate mitochondrial stress adaptation and angiogenesis, while circRNAs, including *circ_0000517*, *circ-MBOAT2*, and *circ-CREBBP*, promote glutamine uptake and α-ketoglutarate production via miRNA-mediated derepression of key metabolic enzymes. In lipid metabolism, lncRNAs such as *PVT1*, *linc-ADAL*, *lncHR1*, and *SLC25A21-AS1* modulate c-Myc activation, SREBP-1c/FASN signaling, and transcriptional scaffolding mechanisms that drive *de novo* lipogenesis and lipid remodeling; while circRNAs, including *circPPP1R12A* and *circZKSCAN1*, influence AKT/mTOR and MAPK pathways to sustain anabolic growth. Redox metabolism and nutrient sensing represent additional layers of regulation. LncRNAs such as *GDIL* and *IDH1-AS1* control GSH levels, α-ketoglutarate production, and HIF1α stability, thereby modulating ROS homeostasis and metabolic adaptation, while *circACC1* functions as a metabolic stress sensor through AMPK complex stabilization, promoting ATP and NADPH homeostasis. Finally, ncRNAs interacting with mTOR and nutrient-sensing pathways, including *NEAT1*, *SLC25A21-AS1*, *circCGNL1*, and *circZKSCAN1*, further illustrate how lncRNAs and circRNAs converge on central signaling nodes that coordinate metabolic plasticity and tumor growth.

These examples illustrate that ncRNAs rarely act as isolated metabolic switches. Instead, they operate within broader regulatory networks shaped by oncogenic signaling, nutrient availability, oxygen tension, and cellular state. Consequently, the metabolic effects attributed to a given lncRNA or circRNA may vary substantially across tumor types, experimental models, and microenvironmental conditions. Many ncRNA–metabolism relationships have been characterized in limited experimental systems, often relying on long-established cancer cell lines and simplified culture conditions. Given the genomic instability and metabolic rewiring typical of these models, their ability to faithfully recapitulate tumor metabolism in vivo requires careful evaluation.

From a translational perspective, one of the most compelling aspects emerging from this review is the integration of ncRNA profiling with metabolomics for early cancer detection and disease stratification. The combined analysis of lncRNA and circRNA signatures has the potential to improve the specificity and sensitivity of cancer diagnostics. Profiling circulating lncRNAs and circRNAs in liquid biopsies may enable early detection of tumor-specific metabolic signatures. Simultaneous monitoring of multiple ncRNA classes allows the capture of complementary layers of metabolic and transcriptional regulation that would be overlooked when focusing on a single ncRNA category.

From a therapeutic standpoint, targeting ncRNAs-driven metabolic axes represents a promising strategy to sensitize tumors to conventional or metabolism-oriented therapies. LncRNA- or circRNA-directed approaches may be combined with inhibitors of glycolysis, glutaminolysis, lipid metabolism, or key signaling nodes such as mTOR and c-Myc to achieve synergistic effects. For instance, inhibition of *circACC1* or *NEAT1* in combination with mTOR-targeted drugs may disrupt both metabolic adaptation and proliferative signaling. On the other hand, lncRNAs such as *PVT1* or *OTX2-AS1* could serve as biomarkers to guide patient stratification and the selection of rational combination therapies. Such combinatorial strategies offer the potential for tumor-specific interventions that maximize therapeutic efficacy while limiting systemic toxicity.

NcRNA-based therapeutic strategies [217], including direct targeting, modulation of ncRNA–protein interactions, and exosome-mediated delivery, represent promising but still evolving approaches. The limited clinical success of therapies targeting single metabolic pathways, such as mTOR inhibition [218,219], further underscores the need for multilayered strategies that incorporate ncRNA-driven regulatory axes [220]. However, clinical translation remains challenging because of the pleiotropic and context-dependent functions of ncRNAs, as well as the need for efficient and selective delivery systems, including nanoparticle-based platforms for siRNA- or shRNA-mediated targeting [221]. Moreover, many studies still rely on correlative analysis, and functional validation in physiologically relevant models is required to establish causal relationships between ncRNAs and metabolic phenotypes [222,223,224].

The increasing recognition of cellular and spatial heterogeneity further complicates interpretation. While lncRNAs often display cell-type- and cell-state-specific expression patterns, metabolic phenotypes vary across tumor regions in response to microenvironmental gradients. This mismatch suggests that ncRNA–metabolism interactions may be spatially restricted, transient, or confined to specific cellular subpopulations, rather than representing uniform regulatory mechanisms across the tumor mass.

Future progress will require integration of ncRNA profiling with quantitative flux-based metabolic analyses and physiologically relevant experimental models, including three-dimensional cultures, patient-derived systems, and single-cell or spatial approaches. Such strategies are essential to move beyond predominantly correlative associations and to define the contexts in which lncRNAs and circRNAs exert functionally meaningful control over cancer metabolism.

Overall, current evidence supports a context-dependent involvement of lncRNAs and circRNAs in cancer metabolic regulation, although their precise functional contribution requires further successful clinical exploitation, which will depend on integrative, pathway-oriented, and quantitatively validated approaches.

## 7. Conclusions

In conclusion, this review provides a pathway-oriented perspective on how lncRNAs and circRNAs converge to regulate cancer metabolic reprogramming. By organizing both ncRNA classes according to major metabolic pathways (glycolysis, glutaminolysis, lipid metabolism, redox control, and nutrient-sensing signaling), we emphasize their coordinated roles within shared oncogenic networks rather than viewing them as isolated regulators. This unified view strengthens the rationale for combining ncRNA profiling with metabolic analyses to improve patient stratification and to design metabolism-oriented combination therapies.

NcRNAs are increasingly recognized as potential contributors to metabolic regulation rather than merely passive correlates of metabolic rewiring. However, defining their spatial and temporal functions will require quantitative and causally oriented approaches, including flux-based metabolic analyses, physiologically relevant models, and single-cell or spatially resolved profiling.

Further functional validation and quantitative metabolic studies will be essential to translate ncRNA-mediated metabolic regulation into clinically actionable precision oncology strategies.

## Figures and Tables

**Table 1:** Distribution of metabolism-associated lncRNAs and circRNAs across major cancer types revealed by this study.

Cancer Type	LncRNAs Identified	CircRNAs Identified	Total ncRNAs
**HCC**	*XIST*, *LINC00152*, *SNHG1*, *OTX2-AS1*, *lncRNA-MIF*, *NEAT1*, *MEG3*, *HUCL*, *PVT1*, *IDH1-AS1*	*circRPN2*, *circZKSCAN1*	**12+**
**Colorectal cancer**	*XIST*, *lncRNA-MIF*, *MEG3*, *GAS5*	*circACC1*, *circ0032821*, *circLRCH3*, *circPDIA3*, *circPDE4D*, *circHIPK3*, *circFBXW7*	**11+**
**Gastric cancer**	*LINC00152*, *SNHG1*, *H19*, *Lnc-SLC2A12-10:1*, *MALAT1*, *LINC00511*, *LINC01534*	*circ_0008450*, *circPGD*, *circSCMH1*	**10+**
**NSCLC**	*XIST*, *IGFBP4-1*, *LETS1*	*circSLC25A16*, *circ_0000517*, *circZKSCAN1*, *circPPP1R12A*	**7**
**Breast cancer**	*H19*, *LETS1*, *LINC00511*, *GAS5*, *MLLT4-AS1*	*circRNF20*, *circFNDC3B*	**7**
**Bladder cancer**	*UCA1*, *CARMN*	*circNR3C1*, *circCGNL1*	**4**
**Pancreatic cancer/PDAC**	*—*	*circ_0067934*, *circ-MBOAT2*, *circSLIT2*, *hsa_circ_0065394*	**4**
**EC**	*PVT1*, *LINC00511*, *MALAT1*, *IQCH-AS1*, *CARMN*, *LINC00648*	*—*	**6**
**Glioma**	*XIST*, *IDH1-AS1*	*circ-CREBBP*	**3**
**NPC**	*—*	*circRNF13*, *circCGNL1*	**2**
**ESCC**	*SLC25A21-AS1*	*—*	**1**

**Table 2:** LncRNAs and circRNAs involved in cancer metabolic reprogramming: pathways, mechanisms, and clinical relevance. LncRNAs and circRNAs implicated in more than one metabolic pathway are highlighted in bold, as well as the main pathways. The arrows indicate increase (↑) or decrease (↓).

Metabolic Pathway	Representative ncRNAs	Main Molecular Mechanism	Metabolic Effects	Cancer	Clinical Relevance
**Glycolysis/** **Warburg effect**	*XIST*	ceRNA activity; activation of PI3K/AKT signaling; upregulation of glycolytic enzymes (e.g., HK2, PKM2, GLUT1); modulation of HIF-1α-dependent transcription	↑ Glucose uptake ↑ glycolytic flux ↑ Lactate production ↑ Warburg effect	Colorectal cancer; HCC; glioma; NSCLC; breast cancer	Oncogenic lncRNA. Associated with metabolic reprogramming, tumor progression, and chemoresistance. Potential prognostic biomarker and therapeutic target.
	*LINC00152*	ceRNA activity; activation of PI3K/AKT/mTOR signaling; upregulation of glycolytic regulators (e.g., GLUT1, HK2); modulation of HIF-1α/c-Myc-dependent transcription	It supports metabolic adaptation and tumor growth: ↑ Warburg effect ↑ glycolytic flux ↑ Lactate production	HCC; gastric cancer	Oncogenic lncRNA. Associated with tumor progression and poor clinical outcomes. Proposed biomarker and therapeutic target in gastrointestinal cancers.
	*SNHG1*	ceRNA activity; derepression of HK2 via miRNA sponging; activation of PI3K/AKT/mTOR and c-Myc signaling; promotion of glycolytic gene expression	↑ HK2-driven glycolysis: ↑ Glucose consumption/uptake ↑ Lactate production	HCC; gastric cancer (including paclitaxel-resistant context);	Oncogenic lncRNA; linked to aggressive behavior and therapy resistance (notably paclitaxel resistance in GC via glycolysis). Potential prognostic biomarker and target to counter metabolic/chemoresistance.
	***OTX2-AS1***	Transcribed antisense to *OTX2*; modulates *OTX2*-associated transcriptional programs; it may sponge tumor-suppressive miRNAs (context-dependent); associated with transcriptional control of metabolic genes	Indirect regulation of oxidative metabolism; potential shift between glycolytic and oxidative metabolic states; enhanced proliferative capacity in glycolysis-dependent tumors	Medulloblastoma (WNT, Group 3, Group 4 subtypes)	High expression correlates with increased sensitivity to BCL-2 inhibitors. Potential predictive biomarker for therapeutic stratification.
	*TRINGS*	Transcriptionally activated by p53 under glucose deprivation; interacts with components of the STRAP–GSK3β–NF-κB pathway	↑ survival under glucose starvation; it maintains metabolic adaptation during nutrient limitation Prevents necrosis in low-glucose conditions	Colorectal cancer; other solid tumors	Stress-adaptive lncRNA. Promotes tumor survival under metabolic stress and may contribute to resistance to nutrient deprivation therapies.
	*UCA1*	miR-145 sponging with upregulation of HK2 and GLUT1 via PI3K/AKT pathway	↑ Glucose uptake ↑ glycolytic flux ↑ Lactate production; metabolic adaptation	Bladder cancer; gastric cancer; breast cancer	Prognostic biomarker; chemoresistance; tumor growth.
	*H19*	miRNA sponging; modulation of c-Myc and HIF-1α signaling	↑ glycolytic flux ↑ Lactate production; metabolic flexibility	Breast cancer; gastric cancer	Chemoresistance-associated lncRNA; prognostic biomarker.
	*Lnc-SLC2A12-10:1*	miRNA sponging (miR-105-5p; miR-150-3p); regulation of SLC2A family glucose transporter expression	Modulation of glucose uptake; regulation of glycolytic flux	Gastric cancer	Circulating exosomal biomarker; potential regulator of metabolic aggressiveness.
	*LncRNA BCRT1*	HIF-1α-dependent transcriptional activation under hypoxia; ceRNA activity via miR-432-5p/CCR7 axis; regulation of miR-1303/PTBP3; modulation of FGF7 expression	Hypoxia-associated metabolic adaptation; support of glycolytic reprogramming and metastatic potential	Breast cancer; cervical cancer; osteosarcoma	Metastasis-associated lncRNA; hypoxia-responsive biomarker; promotion of EMT and metastatic phenotype; potential therapeutic target.
	*lncRNA IGFBP4-1*	Regulation of glycolytic metabolism and ATP production; interaction with JAK/STAT signaling pathways	↑ ATP production; enhancement of Warburg effect	Lung cancer; bladder urothelial carcinoma	Metastasis-associated lncRNA; signaling-dependent oncogenic lncRNA.
	***lncRNA LETS1***	Amplification of TGFβ–SMAD signaling	It promotes metabolic plasticity; enhancement of glycolytic, glutamine, and lipid metabolic shifts during EMT.	Lung cancer; breast cancer	Metastasis-associated lncRNA; reinforcement of EMT-associated transcriptional programs.
	*MALAT1*	Regulation of HIF-1α and c-Myc-dependent transcriptional programs; modulation of glycolytic gene expression (e.g., GLUT1, HK2, LDHA); ceRNA activity affecting metabolic regulators	↑ glycolytic flux ↑ Lactate production; metabolic adaptation during tumor progression	EC; gastric cancer; multiple types of cancers	EMT- and metabolism-associated lncRNA; prognostic biomarker.
	*IQCH-AS1*	ceRNA by sponging tumor-suppressive miRNAs regulating glycolytic enzymes and pro-glycolytic signaling pathways (e.g., HK2, PKM2, LDHA, PI3K/AKT/mTOR axis, depending on tumor context).	It promotes oncogenic metabolic signaling: ↑ Warburg effect ↑ Glucose uptake ↑ Lactate production ↑ cell proliferation and tumor growth	Several solid tumors	High expression correlates with poor prognosis, enhanced tumor aggressiveness, and may serve as a prognostic biomarker and potential therapeutic target.
	*CARMN*	It regulates transcriptional programs and miRNA networks controlling cell cycle and metabolic genes	Downregulation may favor ↑ glycolysis and proliferative metabolism	Colorectal cancer; bladder cancer; others	↓ expression associated with tumor progression and poor prognosis; potential tumor suppressor biomarker.
	*LINC00648*	ceRNA sponging tumor-suppressive miRNAs; may regulate PI3K/AKT-related pathways	Metabolic reprogramming: ↑ glycolytic flux ↑ proliferation ↑ tumor growth	Lung cancer; gastric cancer (context-dependent reports)	High expression associated with poor survival; potential prognostic biomarker.
	*gLINC*	Hypoxia-induced lncRNA; transcriptionally activated by HIF-1α; enhances glycolytic gene expression; may stabilize glycolytic mRNAs and/or reinforce HIF-1α-dependent transcriptional programs	↑ expression of glycolytic enzymes (e.g., HK2, PFK, LDHA context-dependent) ↑ Glucose uptake ↑ Lactate production ↑ Warburg effect ↑ tumor growth under hypoxia	Breast cancer; other hypoxia-driven tumors	It promotes metabolic reprogramming and tumor aggressiveness; potential biomarker of hypoxic metabolic adaptation; candidate target for anti-glycolytic therapies.
	*lncRNA-MIF*	Negatively regulates c-Myc signaling: transcriptionally activated by c-Myc, forming a negative feedback loop; it acts as a ceRNA by sequestering miR-586, thereby relieving miR-586-mediated repression of FBXW7; increased FBXW7 promotes SCF–FBXW7-mediated ubiquitin–proteasome degradation of c-Myc, reducing c-Myc stability and transcriptional activity	↓ glycolytic enzyme expression (e.g., HK2, LDHA, PKM2) ↓ Glucose uptake ↓ Lactate production ↓ c-Myc-driven anabolic metabolism	HCC; colorectal cancer; other solid tumors	Tumor-suppressive lncRNA; ↓ expression associated with tumor progression and poor prognosis; potential regulator of c-Myc-dependent metabolic reprogramming.
	*lincRNA-p21*	Transcriptionally induced by p53; interacts with transcriptional regulators (e.g., hnRNP-K) to repress target genes; inhibits HIF-1α translation and activity; may suppress β-catenin and AKT/mTOR signaling	↓ HIF-1α-mediated glycolytic gene expression (e.g., GLUT1, LDHA) ↓ Glucose uptake ↓ Lactate production ↓ Warburg effect ↑ apoptosis under hypoxia	Colorectal cancer; breast cancer; HCC; lung cancer; others	Tumor-suppressive lncRNA; low expression correlates with poor prognosis; regulates hypoxia-driven metabolic reprogramming; potential biomarker of p53 functional status.
	*circ_0067934*	miR-1324 sponging; activation of Wnt/β-catenin signaling	It promotes glycolytic and proliferative metabolic programs.	Pancreatic cancer	Oncogenic circRNA; tumor progression biomarker.
	***circACC1***	Derived from exons 2–4 of *ACC1* pre-mRNA; under serum deprivation, upregulated via the JNK/c-Jun pathway, favoring circularization; it binds directly to AMPK β1 and γ1 subunits, stabilizing the AMPK complex, preventing ubiquitination/degradation, and enhancing kinase activity; mimics allosteric AMPK activation	↑ AMPK activity ↑ glycolysis ↑ fatty acid β-oxidation ↓ lipid accumulation ↑ ATP and NADPH homeostasis enhanced survival under nutrient limitation	Colorectal cancer	Metabolic stress-responsive circRNA. Supports tumor survival under nutrient deprivation. Overexpression promotes tumor growth, whereas knockdown reduces aggressiveness.
	*hsa_circ_0065394*	Peptide encoding (cPFKFB4); modulation of PKM1/PKM2 alternative splicing via interaction with splicing factors	It promotes PKM2-driven glycolysis; enhancement of Warburg effect under hypoxia.	Pancreatic cancer	hypoxia-associated oncogenic circRNA; metabolic reprogramming driver
	*circMAT2B*	miRNA sponge (notably for miR-338-3p); it relieves repression of PKM2; enhances HIF-1α signaling, forming a positive regulatory loop that promotes glycolytic gene expression	↑ PKM2 expression ↑ Glucose uptake ↑ Lactate production ↑ Warburg effect ↑ tumor growth	HCC; other solid tumors	High expression correlates with poor prognosis; promotes hypoxia-driven metabolic reprogramming; potential target for anti-glycolytic therapy.
	*circSLC25A16*	miRNA sponge (notably for miR-488-3p); derepresses HIF-1α, enhancing its transcriptional activity; promotes expression of glycolytic enzymes	↑ HIF-1α signaling ↑ HK2 and other glycolytic enzymes ↑ Glucose uptake ↑ Lactate production ↑ Warburg effect	NSCLC	High expression correlates with tumor progression and poor prognosis; contributes to hypoxia-driven metabolic reprogramming; potential therapeutic target.
	*circRNF20*	miRNA sponge (notably for miR-487a); it relieves repression of HIF-1α; promotes HIF-1α stabilization and transcriptional activity; enhances glycolytic gene expression	↑ HIF-1α activity ↑ HK2 and other glycolytic enzymes ↑ Glucose uptake ↑ Lactate production ↑ Warburg effect	Breast cancer	High expression associated with poor prognosis; promotes hypoxia-driven metabolic reprogramming; potential target for anti-glycolytic strategies.
	*circ_0008450*	miR-422a sponging; upregulation of SOX4	It promotes glycolytic reprogramming	Gastric cancer	Metastasis-associated circRNA; promotion of EMT-associated metabolic plasticity.
	*circ0032821*	miR-515-5p sponging; activation of SOX9-dependent transcriptional programs	It promotes metabolic plasticity and stress adaptation.	Colorectal cancer	Chemoresistance-associated circRNA; predictive biomarker candidate.
	*hsa_circ_103089*	miRNA sponge for miR-876-5p, thereby relieving repression of EGFR. Activation of the miR-876-5p/EGFR axis enhances downstream oncogenic signaling (e.g., PI3K/AKT/mTOR), promoting glycolytic gene expression	↑ EGFR signaling ↑ glycolytic activity ↑ Glucose uptake ↑ Lactate production ↑ Warburg effect ↑ migration and invasion	NSCLC	High expression associated with malignant progression and cisplatin resistance; potential biomarker of aggressive phenotype and therapeutic response.
	*circNR3C1*	It binds to c-Myc and prevents its interaction with BRD4, impairing formation of the c-Myc/BRD4 transcriptional complex; it reduces transcription of c-Myc target genes involved in metabolic reprogramming	↓ c-Myc-dependent glycolytic gene expression ↓ Glucose uptake ↓ Lactate production ↓ Warburg effect ↓ proliferation and tumor growth	Bladder cancer	Tumor-suppressive circRNA; low expression associated with enhanced metabolic reprogramming and tumor progression; potential biomarker and therapeutic candidate targeting c-Myc-driven tumors.
	*circSCMH1*	miRNA sponge for miR-296-3p, thereby relieving repression of HSPB7. Through activation of the miR-296-3p/HSPB7–GLUT3 axis, circSCMH1 downregulates GLUT3, reducing glucose transport into tumor cells	↓ GLUT3 expression ↓ Glucose uptake ↓ glycolytic flux ↓ Lactate production ↓ Warburg effect ↓ migration and invasion	Malignant tumors (context-dependent; solid cancers).	Tumor-suppressive circRNA; reduced expression associated with enhanced glycolysis and metastatic potential; potential biomarker of aggressive phenotype.
	*circRNF13*	It stabilizes SUMO2 mRNA, increasing SUMO2 expression. Enhanced SUMO2 promotes GLUT1 degradation, thereby reducing glucose transport into tumor cells	↓ GLUT1 protein levels ↓ Glucose uptake ↓ glycolytic flux ↓ Lactate production ↓ Warburg effect	NPC	Tumor-suppressive circRNA; reduced expression associated with enhanced glycolysis and tumor progression; potential biomarker of metabolic aggressiveness.
	*circRPN2*	Dual tumor-suppressive functions: it binds *ENO1*, promoting its ubiquitin-mediated degradation, thereby directly reducing glycolytic enzyme levels; it functions as a miRNA sponge for miR-183-5p, relieving repression of FOXO1, a metabolic tumor suppressor that antagonizes glycolytic signaling	↓ ENO1 protein levels ↓ glycolytic flux ↓ Glucose consumption ↓ Lactate production ↓ Warburg effect ↓ tumor growth and progression	HCC	Tumor-suppressive circRNA; reduced expression associated with enhanced glycolysis and aggressive phenotype; potential metabolic therapeutic target.
	*circFNDC3B*	Encoding a 218-amino-acid peptide (circFNDC3B-218aa). The peptide inhibits the EMT regulator Snail, leading to increased expression of Fructose-1,6-bisphosphatase 1 (FBP1), a key gluconeogenic enzyme that counteracts glycolysis	↑ FBP1 expression ↓ glycolytic flux ↓ Warburg effect restoration of glycolysis–gluconeogenesis balance ↓ EMT and tumor progression	Colon cancer	Tumor-suppressive circRNA; reduced expression associated with enhanced glycolysis and EMT; highlights coding potential of circRNAs as metabolic regulators.
	*circSLIT2*	miRNA sponge (notably for miR-510-5p); it relieves repression of c-Myc negative regulators; suppresses c-Myc signaling and downstream glycolytic transcriptional programs	↓ c-Myc activity ↓ glycolytic enzyme expression (e.g., HK2, PKM2, LDHA) ↓ Glucose uptake ↓ Lactate production ↓ Warburg effect	Colorectal cancer; gastric cancer; PDAC	Tumor-suppressive circRNA; low expression associated with enhanced tumor progression and metabolic reprogramming.
**Glutaminolysis/amino acid metabolism**	***MLLT4-AS1***	It encodes 21-aa micropeptide XBP1SBM; Glutamine scarcity promotes activation of IRE1α–XBP1s axis; ↑ MLLT4-AS1/XBP1SBM, XBP1SBM binds XBP1s, retains it in the nucleus leading ↑ XBP1s occupancy at VEGF promoter	Redox/ER stress signaling mTOR/nutrient adaptation (indirect); ↑ VEGF transcription	Validated in vivo models (solid tumors under metabolic stress).	↓ XBP1SBM translation reduces angiogenesis and tumor growth; highlights therapeutic potential targeting stress-adaptive micropeptides.
	***circ_0000517***	It sponges miR-330-5p; ↑ YY1 (transcription factor); activation of metabolic gene programs	It promotes dual metabolic rewiring (glycolysis + glutaminolysis): ↑ Glucose uptake ↑ Lactate production ↑ ATP generation ↑ Glutamine consumption ↑ Glutamate & α-KG levels ↑ proliferation	NSCLC	Oncogenic circRNA; potential therapeutic target.
	*circ-MBOAT2*	It sponges miR-433-3p ⟶ affecting ↑ glutamic-oxaloacetic transaminase 1	↑ Aspartate production ↑ amino acid availability ↑ TCA cycle intermediates (anaplerosis) ↑ proliferation	Pancreatic cancer	Oncogenic circRNA; potential metabolic vulnerability target.
	*circ-CREBBP*	It sponges miR-375, ↑ glutaminase expression	↑ Glutamine-to-glutamate conversion ↑ α-ketoglutarate production ↑ TCA cycle fueling ↑ tumor growth	Glioma	Oncogenic circRNA; enhances glutamine dependency; potential target in glutamine-addicted tumors.
**Lipid** **metabolism**	*PVT1*	Interaction with c-Myc; regulation of lipogenic transcriptional networks	Enhancement of *de novo* lipogenesis and anabolic metabolism	EC; HCC; other cancers	Oncogenic lncRNA; metabolic vulnerability biomarker; therapeutic target.
	*HUCL*	It sponges miR-9, ↑ PPARα, ↑ ACSL1 activation	It promotes lipid metabolic reprogramming: ↑ fatty acid activation (ACSL1) ↑ lipid accumulation ↑ tumor growth	HCC	Oncogenic lncRNA.
	*LINC00511*	ceRNA activity via sponging miR-765; activation of PI3K/AKT/mTOR and c-Myc signaling; upregulation of SREBP1-driven lipogenic genes (e.g., FASN, ACC); promotion of lipid biosynthesis; interaction with metabolic transcriptional networks	It promotes a lipid biosynthesis-associated metabolic phenotype.	EC; gastric cancer	Oncogenic lncRNA; potential diagnostic and prognostic biomarker associated with tumor progression.
	*LNMICC*	It recruits NPM1 to FABP5 promoter, ↑ FABP5 transcription	↑ fatty acid transport/utilization ↑ metastatic potential	Cervical cancer	Promoting metastasis.
	*linc-ADAL*	It interacts with hnRNPU, ↑ transcription of lipogenic genes (ACLY, ACC, FASN); it binds IGF2BP2, ↑ mRNA stability & translation	It coordinates transcriptional and post-transcriptional control of lipogenesis: ↑ fatty acid synthesis ↑ lipid accumulation ↑ adipogenic differentiation	Metabolic tissues	Primarily regulates adipogenesis; lncRNA metabolic tissue-specific, not tumor-specific.
	***SLC25A21-AS1***	It stabilizes *SLC25A21* mRNA modulating tryptophan metabolism & NAD^+^ production; in nucleus: interacts with NPM1, ↑ c-Myc transcription	↑ Tryptophan catabolism ↑ NAD^+^ biosynthesis ↑ energy metabolism ↑ proliferation, migration, metastasis ↑ cisplatin resistance; redox metabolism/NAD^+^ biosynthesis mTOR/c-Myc signaling (indirect)	ESCC	Oncogenic lncRNA; downregulated by palmitic acid/high-fat diet; potential prognostic biomarker and therapeutic target.
	*lncHR1*	↓ SREBP-1c (lipogenic transcription factor); ↓ FASN expression	↓ fatty acid synthesis ↓ triglyceride accumulation ↓ lipid droplet formation ↓ tumor growth	HCC	Tumor-suppressive lncRNA; loss promotes lipogenic reprogramming; potential metabolic therapeutic target.
**Redox metabolism/ROS control**	*PICSAR AC025176.1* *AC016405.3* *LINC02313* *AP002387.1* *AC004687.1* *AL451069.3*	Regulation of MPT-driven necrosis pathways; transcriptional adaptation under metabolic and oxidative stress; ↓ PD-L1 expression	Altered mitochondrial homeostasis; metabolic plasticity; immune–metabolic crosstalk.	HCC; liver cancer organoids	Prognostic signature; association with immune suppression and therapy resistance.
	*GDIL*	Scaffold function; inhibition of CHAC1-mediated GSH degradation; maintenance of intracellular GSH levels	↑ GSH accumulation ↓ ROS levels; enhanced redox buffering capacity	Colorectal cancer; OC	Biomarker; platinum resistance; chemoresistance; potential therapeutic target.
	*BISPR*	Induced by interferon signaling; positively regulates BST2 expression; modulates type I interferon pathways linking inflammation and metabolic adaptation	↑ adaptation to oxidative stress; ↑ ROS tolerance immune-associated metabolic rewiring	Breast cancer; HCC; others	Associated with tumor aggressiveness and immune infiltration patterns; potential immunometabolic biomarker.
	*lnc IDH1-AS1*	Antisense lncRNA regulating IDH1 expression and activity; modulates NADPH production and TCA cycle flux; influences mitochondrial oxidative metabolism	↓ IDH1 activity (when downregulated) ↓ NADPH production ↑ oxidative stress (ROS accumulation); altered TCA cycle metabolism; impaired redox homeostasis	Prostate cancer; glioma; others	Reduced expression associated with tumor progression; may influence redox balance and metabolic vulnerability; potential metabolic biomarker.
	*circPGD*	miRNA sponging (miR-16-5p/ABL2 axis); peptide encoding (PGD-219aa; PGD/PPP-linked)	Support of NADPH-related redox buffering and anabolic metabolism (PPP-associated).	Gastric cancer	Oncogenic circRNA; promoting tumor growth; potential biomarker/therapeutic target.
	*hsa_circ0071589*	miR-133b sponging; activation of SOX13-dependent transcriptional programs	It promotes metabolic flexibility and redox homeostasis.	Colorectal cancer	Stemness-associated circRNA; chemoresistance biomarker.
	*circPDE4D*	Regulation of signaling pathways linked to stress adaptation and cell survival.	Maintenance of metabolic balance under therapeutic stress.	Colorectal cancer	Tumor-suppressive circRNA; predictor of therapeutic response.
	*circPDIA3*	Interaction with GSDME-C domain; inhibition of pyroptosis	Reduced ROS-mediated cell death; enhanced oxidative stress tolerance.	Colorectal cancer	Oxaliplatin resistance-associated circRNA.
**mTOR/** **nutrient-sensing pathways**	***KB-1460A1.5***	Activation of PI3K/AKT/mTOR signaling; modulation of growth-associated transcriptional programs; support of anabolic metabolism	↑ mTOR-driven anabolic metabolism ↑ biosynthetic activity ↑ proliferation and tumor growth	Context-dependent solid tumors	Putative oncogenic lncRNA; associated with tumor progression; potential biomarker of proliferative/metabolic activation.
	*lnc PCNAP1*	ceRNA activity via miR-154/PCNA/HBV axis and miR-340-5p/ATF7 signaling; derepression of SOX4 in breast cancer; promotion of HBV-associated oncogenic signaling	It supports growth-associated metabolic adaptation.	HCC (HBV-related); breast cancer	Oncogenic lncRNA; tumor progression marker; viral-associated tumorigenesis.
	*lncRNA CDC6*	ceRNA activity via miR-215/CDC6 axis; modulation of DNA replication licensing; interaction with PI3K/AKT signaling networks	Increased biosynthetic demand associated with cell cycle progression.	Breast cancer; prostate cancer	Oncogenic lncRNA; tumor progression marker; potential therapeutic target.
	*LINC01088*	ceRNA activity via miR-22/CDC6 axis; regulation of PI3K/AKT signaling	Activation of growth-associated anabolic pathways.	Prostate cancer	Oncogenic lncRNA; proliferation-associated biomarker.
	*NEAT1*	Paraspeckle regulation downstream of mTORC1; modulation of glycolytic gene expression	Metabolic plasticity; it activates PPARα signaling, ↑ transcription of FAO genes; it sustains ATGL-mediated lipolysis; forms a context-dependent feedback loop with *TP53*	HCC; gastric cancer	Oncogenic lncRNA; regulator of mTOR-associated metabolic adaptation; prognostic biomarker; it supports lipid-fueled tumor growth; interacts with TP53 signaling; potential metabolic therapeutic target.
	***LINC01534***	ceRNA sponging tumor-suppressive miRNAs, activating oncogenic pathways such as PI3K/AKT, converging on mTOR signaling	↑ Glucose uptake ↑ Lactate production ↑ glycolytic enzyme expression	Esophageal cancer; gastric cancer; others	Overexpression correlates with advanced stage and poor prognosis; potential metabolic therapeutic target.
	***LINC00930***	It interacts with chromatin-modifying complexes; epigenetically upregulates metabolic genes; enhances glycolytic transcriptional programs, possibly intersecting with mTOR-driven anabolic signaling	↑ glycolytic flux ↑ tumor growth ↑ metabolic reprogramming	Breast cancer; others	Strong association with poor prognosis; candidate biomarker and potential target for metabolic therapy.
	***MEG3***	It activates p53 signaling; inhibits PI3K/AKT/mTOR pathway; may function as ceRNA for oncogenic miRNAs; regulates transcription of metabolic genes via p53-dependent mechanisms	↓ glycolytic enzyme expression ↓ Glucose uptake ↓ Lactate production ↓ mTOR-driven anabolic metabolism ↑ apoptosis and ROS-mediated stress response	HCC; colorectal cancer; lung cancer; glioma; breast cancer; others	Tumor-suppressive lncRNA; frequently downregulated; low expression associated with poor prognosis; potential therapeutic target.
	***GAS5***	ceRNA for multiple oncogenic miRNAs; inhibits PI3K/AKT/mTOR signaling; modulates glucocorticoid receptor (GR) activity; regulates metabolic gene expression	↓ Glucose uptake ↓ glycolytic enzyme expression (e.g., HK2, PKM2 in certain cancers) ↓ mTOR-mediated anabolic metabolism ↑ apoptosis ↑ sensitivity to oxidative stress	Breast cancer; colorectal cancer; gastric cancer; lung cancer; HCC; others	Tumor-suppressive lncRNA; low expression associated with poor prognosis; may enhance chemosensitivity and therapeutic response.
	*circ-E-Cad*	Peptide encoding (C-E-Cad); EGFR activation via CR2 domain binding; PI3K/AKT pathway stimulation	Activation of growth-associated anabolic metabolism.	Glioblastoma; gastric cancer	oncogenic circRNA; enhancement of proliferative signaling.
	*circPPP1R12A*	It activates oncogenic signaling pathways promoting proliferation and inhibiting apoptosis;	↑ AKT/mTOR pathway activation (context-dependent) ↑ proliferation ↓ apoptosis ↑ tumor growth and metastasis	NSCLC; colon cancer	Oncogenic circRNA; overexpression associated with aggressive phenotype and enhanced tumor progression.
	*circPTV1*	miRNA sponging; activation of growth-associated signaling pathways (e.g., PI3K/AKT)	It promotes proliferative and anabolic metabolism.	Multiple types of cancers	Oncogenic circRNA; tumor progression and metastasis biomarker.
	*circLRCH3*	miR-383-5p sponging; activation of FGF7 signaling and downstream survival pathways	Support of growth-associated metabolic adaptation.	Colorectal cancer	Chemoresistance-associated circRNA under metabolic stress; potential predictive biomarker.
	***circZKSCAN1***	It sponges miR-185-5p, relieving repression of TAGLN2, indirectly enhancing glycolytic flux; it sponges miR-330-5p, upregulating FAM83A, activating MAPK signaling; it encodes a peptide that promotes FBXW7-mediated degradation of mTOR, suppressing oncogenic signaling	↑ MAPK activation ↑ glycolytic flux ↑ proliferation and chemoresistance; ↓ mTOR signaling ↓ anabolic metabolism ↑ sensitivity to sorafenib	Lung adenocarcinoma; NSCLC; HCC	Dual, context-dependent function; chemoresistance in lung cancer and responsiveness to sorafenib in HCC.
	*circCGNL1*	It sequesters IGF2BP3, preventing it from stabilizing oncogenic mRNAs; when it is downregulated, IGF2BP3 is released, leading to AKT and mTOR phosphorylation, promoting proliferation and suppressing apoptosis	Reduced anabolic signaling: ↓ AKT/mTOR ↓ proliferation ↑ apoptosis	NPC	Tumor-suppressive circRNA; low expression associated with enhanced AKT/mTOR signaling and aggressive tumor behavior.
	*circHIPK3*	miRNA sponging; modulation of AMPK and growth-related signaling pathways	Inhibition of proliferative metabolism.	Multiple types of cancers	Tumor-suppressive circRNA; induction of apoptosis; potential therapeutic target.

## Data Availability

No new data were created or analyzed in this study.

## Abbreviations

The following abbreviations are used in this manuscript:

ACC1	Acetyl-CoA carboxylase 1
ACLY	ATP-citrate lyase
ceRNAs	Competitive endogenous RNAs
circRNA	Circular RNA
dsRNA	Double-stranded RNA
EC	Endometrial cancer
EMT	Epithelial-to-mesenchymal transition
ENO1	α-Enolase
ESCC	Esophageal squamous cell carcinoma
EVs	Extracellular vesicles
FBP1	Fructose-1,6-bisphosphatase 1
*GDIL*	*GSH degradation-inhibiting lncRNA*
*gLINC*	*GlycoLINC*
GSH	Glutathione
HCC	Hepatocellular carcinoma
HIF1α	Hypoxia-inducible factor 1α
HK2	Hexokinase 2
*HUCL*	*Highly upregulated in liver cancer*
IDH1	Isocitrate dehydrogenase
IGF2BP3	Insulin-like growth factor 2 mRNA-binding protein 3
linRNAs	Linear mRNAs
lncRNA	Long non-coding RNA
LNMICC	Lymph node metastasis in cervical cancer
MIF	c-Myc inhibitory factor
miRNAs	MicroRNAs
MPT	Mitochondrial permeability transition
mRNAs	Messenger RNAs
MSI	Mass spectrometry imaging
mTOR	Mammalian target of rapamycin
mTORC1	mTOR complex 1
ncRNA	Non-coding RNA
NPC	Nasopharyngeal carcinoma
NPM1	Nucleophosmin 1
NSCLC	Non-small cell lung cancer
OC	Ovarian cancer
*OTX2-AS1*	*Orthodenticle homeobox 2 antisense RNA*
PDAC	Pancreatic ductal adenocarcinoma
PKM	Pyruvate kinase, muscle type
RBPs	RNA-binding proteins
ROS	Reactive oxygen species
siRNAs	Small interfering RNAs
snRNAs	Small nuclear RNAs
TNBC	Triple-negative breast cancer
*TRINGS*	*Tp53-regulated inhibitor of necrosis*
